# Accelerating technical change through ICT: Evidence from a video-mediated extension experiment in Ethiopia

**DOI:** 10.1016/j.worlddev.2022.106089

**Published:** 2023-01

**Authors:** Gashaw T. Abate, Tanguy Bernard, Simrin Makhija, David J. Spielman

**Affiliations:** a International Food Policy Research Institute, Addis Ababa, Ethiopia, Washington DC, USA; b Bordeaux School of Economics, University of Bordeaux, Bordeaux, France; c International Food Policy Research Institute, Kigali, Rwanda; d International Food Policy Research Institute, Washington DC, USA

**Keywords:** Agricultural extension, ICT, Video-based extension, Crop management, Ethiopia

## Abstract

Despite enthusiasm around applications of information and communications technologies (ICTs) to smallholder agriculture in many lower-income countries, there are still many questions on the effectiveness of ICT-based approaches. This study assesses the impacts of video-mediated agricultural extension service provision on farmers’ adoption of improved agricultural technologies and practices in Ethiopia using data from a two-year randomized experiment. Our results show that the video-mediated extension approach significantly increases uptake of recommended technologies and practices by improving extension access and farmer knowledge. Specifically, we find that video-mediated extension reaches a wider audience than the government’s conventional extension approach and leads to higher levels of farmer understanding and uptake of the subject technologies in those locations randomly assigned to the program. While our results also point to greater extension access and greater knowledge among female spouses in locations where both male and female spouses were targeted by the program, we do not find clear evidence that a more inclusive approach translates into higher uptake of the subject technologies. Finally, we find that the video-mediated approach becomes less costly as the scale of operation increases.

## Highlights

• A video-mediated extension approach introduced in Ethiopia increases technology uptake by improving extension access and farmer knowledge.
• While the video-mediated extension approach improves extension access and knowledge among female spouses, it does not lead to higher uptake rates.
• The video-mediated approach becomes less costly as the scale of operation increases.

## 1 Introduction

The use of information and communications technologies (ICTs) to address a wide array of development issues has gained considerable attention among governments, practitioners, and researchers in recent years (Lwoga & Sangeda, 2019). While early studies focused on mobile phones and text messaging, that attention is quickly shifting to other media, including video. Studies on the video medium tend to explore the pathway leading from increase access and consumption of information to changes in behaviors that result in welfare-improving outcomes. Chong and Ferrara, 2009, Jensen and Oster, 2009 demonstrate that exposure to TV soap operas featuring strong women and smaller families led to reduced fertility and increased women’s autonomy. Berg and Zia, 2013, Banerjee et al., 2018 rely on videos purposefully designed to convey specific messages on issues such as financial literacy or HIV prevention. Bernard et al. (2014) shows that screening short documentaries featuring rural individuals who improved their life outcomes through perseverance and hard work led to significant changes in viewers’ perceptions and future-oriented behavior. Similarly, Riley (2022) shows that screening an inspirational movie to secondary school students containing a locally relevant theme and a strong role model significantly improved educational attainment.

The video medium offers several advantages over many other ICT-based information dissemination approaches. First, videos can be tailored and customized to address localized information needs and context. Several studies demonstrate the importance of locally relevant information, drawing attention to evidence from studies in the economics on education (Jensen, 2012), entrepreneurship (Jensen, 2010) and agriculture (Hanna et al., 2014). Psychologists similarly find a positive relationship between locally relevant information and public health (Bull et al., 1999, Marcus et al., 1998), weight gains (Campbell et al., 1994), smoking habits (Prochaska et al., 1993, Shiffman et al., 2000), and education (Kim & Keller, 2008). Second, videos can encourage role model effects. Role models who are similar to individuals across multiple dimensions of character or identity can encourage them to receive, accept, and internalize messages that lead to desirable changes in behavior (Bandura, 1977, Bandura, 1986). Video-based content can provide exposure to role models, substituting for the individual’s experience or the experience of actual peers while framing messages to promote attitude and behavior change (Bernard et al., 2014). Third, videos can allow for consistent content delivery, thereby reducing errors in conveying sensitive and detailed technical information and countering the adverse effects of unmotivated agents. This can be particularly important when learning about complex behaviors or practices (Barrett et al., 2021, Hanna et al., 2014). Fourth, videos can be produced at a relatively low fixed cost, which increases the approach’s cost effectiveness as the number of viewers increases. Whether used alone or in tandem with other approaches to information dissemination, video can be a powerful medium.

Our study explores these issues in the context of smallholder agriculture in lower-income countries with a basic question: does video-mediated agricultural extension lead to increased and sustained uptake of agricultural technologies and practices by smallholder farmers?^1^ We explore these questions in the context of a large-scale rollout of a video-mediated extension approach by the Government of Ethiopia and Digital Green over a two-year period (the main production seasons in 2017/18 and 2018/19). Our study further asks whether video-mediated extension targeted at both spouses of a household is more effective than when only targeted at the (typically male) household head. We also provide insights into the mechanisms driving the observed effects, and an analysis of the cost-effectiveness of the video-mediated approach with respect to promoting the adoption of pre-determined agricultural practices. Although our study is primarily designed to assess the effect on adoption, we also investigate the resulting effects of adoption on farmers’ yields. Importantly, our study is integrated into the government’s efforts to assess existing extension systems program reforms; and is thus a directly policy-relevant evaluation.

To the best of our knowledge, few studies have sought to measure the effectiveness of videos in promoting agricultural technologies with any degree of rigor or at any sizeable scale (see periodic reviews by Spielman et al., 2021, Fabregas et al., 2019, Nakasone and Torero, 2016, and Aker (2011)). Vasilaky et al. (2015) assessed the effectiveness of a video-mediated approach to promoting the system of rice intensification (SRI) among smallholder farmers in Bihar, India, and found that the approach increased the probability of adoption by 5 percentage points among those who viewed videos, which translated to a 50 percent increase over the mean of their control group. Hörner et al. (2019) assessed the effect of decentralized agricultural extension services and a video intervention on the adoption of integrated soil fertility management in Ethiopia; but found no marginal gains in adoption resulting from the video intervention. Van Campenhout et al. (2021) experimented with video-based agricultural extension in Uganda and found significant effects on maize farmers’ knowledge, input use, and technology adoption. Dzanku et al. (2022) found significant effects for an intervention combining video documentaries and radio listening clubs on the uptake of *rhizobium* inoculation and legume yields in Ghana but were unable to disentangle the effect of the two interventions.

Our study extends this work with new evidence on the effectiveness of the video medium not only to convey information to farmers, but also to advance gender-relevant augmentations to public extension service provision. In doing so, we shift the focus of inquiry from small experiments to a large-scale evaluation of a public program designed to promote the adoption of agricultural technologies and practices (Stevenson et al., 2019, Deaton, 2010, Banerjee and Duflo, 2009). We also shift attention from the role of ICTs in accelerating the adoption of discrete technologies—new varieties or inorganic fertilizers—to more complex management practices, and from conventional input-targeting models to more complex multi-object learning models (Barrett et al., 2021, Banerjee et al., 2019, Hanna et al., 2014). Further, our study explores both learning externalities (Conley and Udry, 2010, Foster and Rosenzweig, 1995) and dis-adoption dynamics (Barrett et al., 2021) by virtue of its multi-year duration, similar to Oyinbo et al. (2022), who also studied the sustained impact of site-specific nutrient management recommendations provided through digital support tools in Nigeria. Our study also introduces a gender dimension to shed light on knowledge accumulation and decision-making within smallholder agricultural households (e.g., Hoel et al., 2021, Palacios-López and López, 2015; Doss, 2015).

Finally, our study expands the emerging literature on the role of ICTs in developing-country agriculture. To date, most studies in this literature have focused on evaluating simple, low-cost text and voice messaging services provided to farmers over mobile networks, and more often for price-related information (Spielman et al., 2021, Camacho and Conover, 2019, Ogutu et al., 2014). Fewer studies examine the role of ICTs in the provision of production-related information and advice. Exceptions include the use of short message services containing information on crop management advice and weather forecasts in India (Fafchamps & Minten, 2012); integrated pest management practices in Ecuador (Larochelle et al., 2017); agronomic advice in India (Cole & Fernando, 2014) and Kenya (Casaburi et al., 2014); radio and mobile phone services to promote certified seeds and fertilizer in Senegal (Voss et al., 2021); animated videos on post-harvest management in Burkina Faso (Maredia et al., 2017); insecticidal neem use in Benin (Bello-Bravo et al., 2018); interactive crop advisory services via mobile phones in India (Fu and Akter, 2016); targeted/site-specific nutrient management recommendations through digital support tools in Nigeria (Arouna et al., 2021, Oyinbo et al., 2022) and Ethiopia (Ayalew et al., 2022); and the video-based interventions noted earlier. Results from these studies vary from no effects of the ICT-based approach on technology adoption, production, or yields (e.g., Fafchamps and Minten, 2012, Voss et al., 2021) to significant changes in input and technology use and yields/profits (e.g., Cole and Fernando, 2014, Arouna et al., 2021, Oyinbo et al., 2022, Ayalew et al., 2022), suggesting the importance of replication to appropriately reflect the context specificity of each intervention.

Our results demonstrate that the video-mediated extension approach led to increases in the uptake of key agricultural technologies and practices that were recommended by public extension providers, rural administrators, and researchers. In the first year of the experiment, we find a 6 percentage points overall increase in farmers’ uptake of the recommended technologies, which translates into a 10 percent increase over the mean of the control group. Across technologies, we find that the video-mediated approach resulted in a 13, 20 and 15 percent increase in uptake over control group means for row planting, precise seeding rate, and urea top/side dressing, respectively. These results endure in the second year of the experiment, pointing to farmers’ effective uptake of the technologies beyond a mere trial in one production season. From these effects, we compute the marginal cost-effectiveness of the video-mediated approach under experimental and scale-up (“full saturation”) scenarios. The cost of each additional adoption under the experimental scenario ranges from USD 16 – 30. However, these figures decrease to USD 3 – 6 when we assume that the video-mediated approach is extended to all *kebeles* (village clusters) in the treatment *woredas* (districts).

Exploring the mechanisms that explain these adoption effects, we find that the video-mediated extension approach led to an increase in extension reach, with a 35 percent increase in farmers’ attendance to extension sessions likely due to increased interest by farmers in the medium. In turn, we find a higher level of knowledge—greater technical understanding of the subject technologies and practices—among farmers in *kebeles* randomly assigned to the video-mediated extension approach. While our results also point to greater participation and knowledge gains among (typically female) spouses who also participated in the video-mediated extension approach, we do not find clear evidence that targeting both spouses translated into higher technology uptake rates. Lastly, we do not find clear evidence that the increased adoption of the technologies promoted by the Ethiopian public extension system translated into higher yields. These null yield results may be related to issues of measurement error, insufficient adaptation of the technologies to context, or other factors discussed later.

The remainder of this paper is organized as follows. Section 2 provides background to the evaluation. Section 3 presents the experimental design of the study including the interventions and treatment arms, timing, sampling, and experimental integrity. Section 4 discusses the empirical strategy, followed by results of the intervention’s primary impacts in Section 5. Section 6 provides an analysis of the intervention’s cost-effectiveness, while Section 7 concludes with policy implications of the findings, avenues for future research, and concluding thoughts.

## 2 Background and context

Ethiopia’s agricultural extension system—one of the largest in Africa in terms of personnel and coverage— has undergone both small experiments and large reforms during the past three decades (Davis et al., 2010). A pillar of these reforms has been the large increase in the number of agricultural extension agents—known locally as Development Agents (DAs)—deployed to advise farmers. During the past 10–15 years, approximately 90,000 DAs have been trained and 18,000 Farmer Training Centers (FTCs) constructed to support these efforts. Recent estimates from the Ministry of Agriculture indicate that about 72,000 DAs are on duty throughout the country, which is roughly-one DA for 235 farm households, making Ethiopia’s extension agent-to-farmer ratio one of the highest in the world.^2^ DAs reportedly reach more than 75 percent of farm households in Ethiopia (CSA, 2017), and every *kebele* is host to an average of three DAs, each with his or her own technical specialization and reporting up to the *woreda* level.^3^

Yet, it is often difficult to draw a robust causal link between an extension system’s size and approach and its outcomes on farming practices, such as technology adoption, and productivity growth. Prior studies on Ethiopia suggest a somewhat ambiguous link between the extension system’s size and approach, and farmer outcomes such as technology adoption, productivity growth, and poverty reduction (Dercon et al., 2009, Spielman et al., 2010, Krishnan and Patnam, 2014, Abay et al., 2018). Specifically, studies of Ethiopia’s extension system tend to suggest only a weak relationship between the technical support provided by DAs to farmers and productivity growth—yield effects are more likely a function of extension’s role in supplying physical inputs such as inorganic fertilizers and improved cultivars than improving farmers’ awareness, understanding, and ability to innovate and adapt with better farming practices, marketing tactics, and risk management strategies (Berhane et al., 2018, Dercon et al., 2009). While these findings may seem surprising given the scale and reach of Ethiopia’s extension system, a deeper analysis of the system suggests that this is entirely plausible given the organizational culture, daily practices, technical and functional skills of DAs, and professional incentives facing Ethiopia’s extension system (Leta et al., 2017, Davis et al., 2010, Kassa, 2002).

For instance, despite multiple changes in the extension approaches and methods over the years, the role of DAs has not changed substantially. DAs continue to be involved not only in providing advice and training to farmers, but also in estimating seed and fertilizer requirements, estimating crop production, and other responsibilities less directly associated with extension service provision (Kassa, 2003). Berhane et al. (2018) estimates that only about 35–50 percent of DAs’ working hours are spent on training and advising farmers, while the remainder of their time is spent on activities such as supplying inputs, managing loan repayments, collecting taxes, mobilizing communities, and supervising rural road maintenance.

In an attempt to improve the extension system’s effectiveness and shift from a “mass campaign” that emphasizes the adoption of physical inputs to a more customized, knowledge-driven service provision system, Digital Green and the Government of Ethiopia piloted a new digitally enabled approach to extension in 2014. The approach builds on a participatory, video-mediated model first developed by Digital Green in India (Gandhi et al., 2009). In its application to Ethiopia, the approach aimed to increase the adoption of suites of agricultural technologies and practices.^4^ An early assessment of Digital Green’s approach in Ethiopia, based on monitoring data from the pilot phase, suggests considerable potential in the approach, particularly in its ability to provide localized examples of farmers’ uptake and application of the technologies or practices, and reach women farmers (Bernard et al., 2016).

Based on the strengths of the pilot phase results, Digital Green expanded its operations in 2017/18 to 68 *woredas* in Ethiopia’s four most agriculturally important regional states.^5^ This scaling-up effort—and the implementors’ collective willingness to host a cluster-randomized controlled rollout—offered an opportunity to generate rigorous and policy-relevant evidence on the effectiveness of Digital Green’s video-mediated extension approach.

Digital Green’s expansion also opens the door to consideration of the gender dimensions of extension and advisory services. The specialization of labor along gender lines in agricultural households is often used to justify targeting the dissemination of certain technologies to men (e.g., production technologies for cereal crops) and others to women (e.g., nutrition and health-related technologies). However, there is plenty of evidence to suggest that few technologies and practices can be reduced and assigned as “male” or “female” for a given household. There is also ample evidence suggesting that adoption of many technologies, whether related to agriculture or nutrition, is more an outcome of intra-household decision-making processes (e.g., Udry, 1996, Doss and Morris, 2001, Hoel et al., 2021). Such processes are, in turn, influenced by the extent to which spouses have access to similar information. Thus, targeting information to one spouse only may contribute to lower-than-optimal adoption rates if the non-targeted spouse does not have the same level of information.

At this stage however, the literature on the potential impact of increasing women’s access to extension services remains weak. In a recent paper, Doss (2015) revisits the argument that the social rates of return on investments in agricultural development are higher when those investments are targeted to women. Reviewing prior empirical studies, Doss (2015) finds only meager evidence to support these claims, not the least because none of the supporting studies rely on convincing identification strategies in their empirical specifications, in turn implying that the results are best interpreted as correlations but not causal relationships. Instead, she suggests that research should focus on identifying where the best returns to investments are found by relying on gender disaggregation as useful analytical categories since farming and food preparation are deeply gendered activities.

In Ethiopia, because women play an important role in agriculture, there is considerable scope to study the interaction between extension agents and women farmers. Women—not just women who head their own households but also women who are part of male-headed households—are potentially central to the adoption of new technologies and practices. For instance, Palacios-López and López (2015) estimate that women contribute 29 percent of the agricultural labor force in Ethiopia. Yet numerous studies also point out that Ethiopian women have had historically limited access to extension services (Mogues et al., 2019, Ragasa et al., 2013; Buchy & Basaznew, 2017).

## 3 Experimental design and data

### 3.1 The intervention and experimental design

To assess the effect of video-mediated extension on our outcomes of interest, we compare how farmers respond to the same information on selected technologies when disseminated through two different approaches: the conventional “chalk-and-talk” extension approach^6^ and the video-mediated approach. The study was designed as a three-arm stratified randomized controlled trial, clustered at the *kebele* level; and was implemented during the 2017/18 and 2018/19 *meher* (rainy) seasons. Clusters were defined at the *kebele* level since it is the primary level at which agricultural extension is organized in Ethiopia. Within each *woreda*, *kebeles* were randomly allocated to one of three groups:

(1) A control group (denoted “Control”) in which the government’s conventional extension approach was implemented with its standard targeting of the (typically male) household head^7^;
(2) A treatment group (denoted “Video”) in which Digital Green’s standard video-mediated approach (described above) was targeted to the (typically male) household head; and
(3) A treatment group (denoted “Video + Spouse”) in which Digital Green’s standard video-mediated approach was targeted at both the (typically male) household head and his/her spouse.^8^

With this design, we are able to test the impact of the video-mediated approach on our outcomes of interest for any household that participated in the treatment (T1 + T2), and the distinct treatments (T1, T2) separately.

In each group, the same suite of agricultural technologies was promoted to farmers using the video-mediated extension approach in the treated *kebeles* and the conventional extension approach in the control *kebeles*. The homogeneous content promoted in the treated and control *kebeles* ensures that we can evaluate the *medium* used for promotion rather than the content itself. The set of promoted technologies—row planting, precise seeding rates, and urea top/side dressing—for Ethiopia’s three main cereal crops—teff, wheat, and maize have been topics of considerable research in Ethiopia (see Appendix A for details), and while several are relatively novel (e.g., row planting and precision seeding for teff and wheat), others have been a standard part of the extension messaging (e.g., maize row planting) for at least two decades.

The intervention was rolled out by Digital Green, the Ministry of Agriculture (MoA), the bureaus of agriculture in each regional state, and local extension staff at both the *woreda* and *kebele* levels. The intervention itself comprises three interlinked components.

*1. Video production:* The cornerstone of Digital Green’s approach is the production of video content that is customized to local context, in that it features local farmers. Digital Green works with partners—subject matter specialists from the *woreda* extension office, DAs from a nearby *kebele*, and model farmers—to produce short videos on selected technologies.^9^ The videos all feature farmers from the locality speaking in a local language while applying the given technology on their farm. Each video is 10–15 min long and designed to address a specific aspect of the technology, often at a specific time in the crop calendar, for example, when land preparation, seeding and basal fertilizer application, and weed management activities are undertaken. The information contained in these videos are those recommended by the MoA and the regional bureaus of agriculture and are often products of research conducted by the Ethiopian Institute of Agricultural Research and regional agricultural research institutes. Content is further systematically customized to local conditions, which may not always be the case in conventional extension where DAs are specialized by training as either crop, livestock/animal husbandry, or natural resource management experts, although they are required to provide extension advices on all of these topics. With heavy workload throughout the year, individual DAs efforts to acquire knowledge and adapt it to localized conditions can be constrained. In this context, locally customized video content is sought to provide non-specialized DAs with locally relevant support to their advice to farmers. While in principle DAs in the control *kebeles* can tailor the content they deliver to farmers and use lead/model farmers to leverage role model effects, they primarily convey only general messages due to capacity and resource constraints. Customizing content to local conditions and delivering such messages to each farmer group in a consistent and standardized manner is widely recognized as unfeasible for the individual DA specialized on a given topic and following the conventional “chalk-and-talk” approach.

*2. Video screening.* DAs screen the videos to farmers during regular extension sessions organized with *kebele* development groups. Development groups are semi-formal administrative structures within each *kebele* that comprises 25–30 farm households and are designed to provide community members with access to extension services and serve as a grassroots forum to discuss local development issues. DAs assigned to a given *kebele* have access to these development groups as part of their day-to-day work. The screenings are conducted with USB-charged PICO projectors and are structured in a manner designed to facilitate effective learning and discussion. Specifically, DAs screen the videos several times during the meeting, and pause the videos at certain points to answer questions or provide additional details. DAs also augment their facilitation with input from model farmers belonging to the development group(s).

In both treatment and control *kebeles,* extension sessions are open to all farmers, organized with development group members, include group discussions, and are conducted several times during the season, each time with new content that is synched with the crop calendar. In both groups, DAs further provide information to farmers through farm visits. Thus, farmers in treatment and control *kebeles* are exposed to similar messages displayed with similar intensities and similar means, except for the reliance on video screenings during the group sessions in the treatment groups.

*Performance monitoring*. Digital Green and MoA closely monitor both DA activities and farmer uptake of what they describe as “non-negotiable” elements of the technology package that must be adopted to achieve success. This is managed through Digital Green’s “Connect Online Connect Offline” (COCO) platform, an open-source customer relationship management system in the treatment *kebeles*. The COCO system provides its users with a back-end data and analytics infrastructure by integrating field-based data collection interfaces designed for low-connectivity environments with databases and dashboards for monitoring program performance. Although the COCO system is not well-aligned with the needs of a program evaluation such as the one described here, it does provide a basic indication of program coverage that can inform more rigorous evaluation activities (Makhija et al., 2019). DAs activities and adoption of technologies by farmers is monitored following a comparable paper-based process in control *kebeles*.^10^ Table 1 summarizes the experimental design and the variation in intervention by treatment status.

**Table 1:** Experimental design and interventions.

Intervention	Treatment status		
	Conventional extension approach (Control: T0)	Video-mediated extension approach (Video: T1)	Gendered video-mediated extension approach (Video + Spouse: T2)
Source of content	MoA	MoA	MoA
Delivery method	Verbal instruction	Video-mediated (repeated video screenings plus group discussions)	
Embeddedness into local context	Uncertain	High	High
Trainer	DAs	DAs + videos featuring peer farmer	
Target group	Household heads (typically male)	Household heads (typically male)	Household heads (typically male) and (typically female) spouses

Table B1 in the appendix shows the timeline of the intervention rollout and our accompanying study. The interventions took place during the main production season (June–August) in both years. A rapid assessment of the intervention rollout and implementation process was conducted in mid-2017 to ensure compliance with the design described above. The first household survey was conducted in January–March 2018 (hereafter *year 1 survey*), following the harvest of all three crops to measure the immediate outcomes of the intervention. A second household survey was conducted in January-March 2019 (hereafter *year 2 survey*) to measure the persistence of these outcomes and spillover effects to non-treated groups in treated *kebeles*. These surveys were complemented by (1) qualitative data collection and analysis at multiple points during the study to better understand the mechanisms underlying expected and observed effects, highlighted by key informant interviews, focus group discussions, and in-depth interviews with farmers, DAs, and extension system officials; and (2) a series of surveys on DA characteristics, incentives, activities, and performance.

## 4 Sampling and data

Sampled *woredas*, *kebeles*, and households were selected using a four-stage sampling process. We purposefully selected 30 *woredas* for the study based on three criteria: (i) *woredas* that were not saturated or fully covered by Digital Green prior to the 2017/18 *meher* season; (ii) *woredas* that Digital Green planned to expand into in that same season; and (iii) *woredas* that would not be fully saturated during the 2017/18 expansion to ensure the presence of within-*woreda* control *kebeles*. *Woredas* with less than nine potential expansion *kebele*s for the 2017/18 *meher* season were excluded from the study. Within each *woreda*, selected *kebeles* were randomly allocated to one of the three study arms such that each arm contained an equal number of *kebeles*.

Even though the *kebele* is the lowest administrative unit in Ethiopia, it typically comprises several development groups. Given the limited number of PICO projectors available for video screenings, it was infeasible for DAs to reach all development groups in a *kebele* with the video-mediated approach. To prevent placement bias in favor of development groups closest to the Farmer Training Center (typically located at a center point in the *kebele*), DAs were instructed to focus their effort on 10 Development Groups spread across the *kebele*: five randomly selected from among the closest Development Groups (where distance to the FTC was less than the *kebele* median), and five randomly selected from among Development Groups located further away (where distance to the FTC was greater than the median).

Seven households were then randomly selected from each *kebele*: 2 from the closest treated Development Group, 2 from the furthest Development Group, and 3 from the Development Group situated at the median distance from the FTC. The random selection of households for the survey followed the same procedure in both treatment and control *kebele*s, thereby ensuring comparability of farmers across groups.^11^

A total of 2,450 farm households were randomly selected from 30 *woreda*s and 350 *kebele*s located in the study area, of which 2,422 households in 347 *kebeles* were interviewed during the first year. In the second year, 2,345 (97 percent) of these households were re-surveyed. For sake of comparability, we focus our analysis on those households who were surveyed in both years (Table 2).^12^

**Table 2:** Sample size, by survey round.

Sample	Video treatment group (T1)	Video + Spouse treatment group (T2)	Control group (T0)	Total
Total number of *woreda*s	30	30	30	30

Total number of *kebele*s				
*Year 1*	115	116	116	347
*Year 2*	112	115	115	342

Total number of households				
*Year 1*	798	812	812	2,422
*Year 2*	764	789	792	2,345

*Note*: Random assignment of *kebeles* to treatment and control groups was stratified by *woreda*. This implies that each of the 30 *woredas* selected for the study contained *kebeles* assigned to both treatment groups and the control group. For this reason, a total of 30 *woredas* are shown in the last column.

The random assignment of treatment arms generated comparable treatment and control groups at different levels—between *kebeles*, and among both household heads and their spouses (Appendix C: Table C1 and C2). We further examine Digital Green and DA compliance with the experimental design by assessing whether videos were screened in the development groups selected for treatment, and not screened in those selected for controls (Appendix C: Table C3). We find that videos were screened in 85 percent (83 percent) of *kebeles* in the pooled treatment group in year 1 (year 2), while a negligible number of farmers in the control group reported having participated in the video-mediated approach.^13^

Household data were collected using separate questionnaires for household heads and spouses. The household head questionnaire covered topics including household characteristics, savings and assets, access to services, technology adoption (further defined below), knowledge of agricultural practices, experience with the video-mediated approach, non-farm income, food security, shocks, crop sales, and plot-level information on land use, production, and inputs. The spouse questionnaire contained similar questions on assets, technology adoption, knowledge, and experience with the video-mediated approach.

## 5 Empirical approach

### 5.1 Outcomes of interest

Our primary outcomes of interest are farmers’ uptake of the subject technologies in the 2017/18 *meher* season and their continued adoption in the 2018 season. Additionally, we look at the following outcomes to understand the mechanisms driving these outcomes: (i) access to extension services and advice from DAs; and (ii) farmers’ awareness and understanding of the subject technologies. We are further interested in (iii) variations in these outcomes resulting from the distinct gender-targeting strategies used in the two treatment arms. Finally, we investigate (iv) whether enhanced adoption of the promoted technologies translates into yield gains.

We measure uptake of the subject technologies based on participants’ responses to questions in the household survey. The question for each crop and technology was structured as follows: “During *meher* 2017/18 has anyone tried [technology *x*] on your farm?” For the sake of brevity, we report the uptake of these technologies without specific reference to the crop being cultivated, although crop-disaggregated results are provided in the appendices.

In Table 3, we report a set of basic statistics on farmers’ uptake of the promoted technologies. Overall, 80 percent of farmers in the control group and 85 percent in the pooled treatment group tried at least one of the technologies over the two-year period. Between these two groups, we do not find differences in uptake rates between those who trialed the technology in year 1 and then reverted to their prior practices in year 2, nor do we find differences between those who trialed of the technology in year 2 but not in year 1. Rather, the relatively higher uptake rate in the treatment group is primarily accounted for by farmer uptake two years in a row: 49 percent in the pooled treatment group compared 44 percent in the control group. These figures provide our first clue that the video-mediated approach may have a sustained effect on farmer uptake and adoption—a point that we investigate in the next section.

**Table 3:** Uptake trends by treatment group (percentage of total in each respective treatment group).

			Row planting		Precise seeding rate		Urea top/side dressing		Any technology	
			Year 1 (%)							
			*No*	*Yes*	*No*	*Yes*	*No*	*Yes*	*No*	*Yes*
Video treatment (T1)	Year 2 (%)	No	40	15	31	22	31	21	14	20
		Yes	13	32	20	26	15	34	17	50
Video + Spouse treatment (T2)		No	41	14	33	22	30	22	16	19
		Yes	12	32	20	25	15	33	17	49
Pooled treatment (T1 + T2)		No	41	14	32	22	30	21	15	19
		Yes	13	32	20	25	15	33	17	49
Control (T0)		No	46	14	39	21	37	17	21	20
		Yes	11	29	20	19	15	31	16	44

*Source*: Authors’ calculations.

We then measure farmers’ access to extension services and advice from DAs in several straightforward and consistent ways. First, we measure access to extension using farmers’ responses to the question, “Has a DA directly provided you with advice/training on [technology *x*]?” for each of the three focus crops. To measure the intensity of access, we also look at the number of times a DA provided advice for a specific crop.

We measure knowledge as the score from a set of questions about the subject technologies that were asked of participants in the household surveys. These questions were drawn directly from the list of “non-negotiable” elements of the subject technologies as set forth by the Ethiopian government’s own technical recommendations, and as incorporated into both the video and the non-video extension materials used by DAs. The knowledge tests were crop-specific, and were made up of 17 questions on teff, 16 on wheat, and 16 on maize. For each respondent, the number of correct responses were totaled and divided by the total number of questions for a given crop yielding a percentage score.^14^

Lastly, we measure yield. We take a cautious approach to measuring yield, recognizing that while this outcome variable is widely used in the literature, it is extremely sensitive to measurement method (Abay et al., 2019). As such, we use three different measures: self-reported harvest quantity divided by the self-reported area under cultivation at the household level; self-reported harvest quantity divided by the self-reported area under cultivation at the plot level; and self-reported harvest quantity divided by plot area, which was calculated by enumerators walking each plot perimeter using GPS devices.^15^

### 5.2 Estimation strategy

Our estimation strategy relies on an intent-to-treat (ITT) approach, which captures the effect of being randomly allocated to a *kebele* where the video-mediated extension approach was introduced, regardless of whether the household member(s) actually participated in a video screening. To estimate these ITT effects, we include all sample households—whether or not they were actually “treated” (i.e., received extension services)—in our analysis, since extension meetings are open to all development group members. Thus, we are estimating the intervention’s effect on the group for whom it was intended.

We restrict our analysis to ITT estimates for two reasons. One is statistical. To estimate the Treatment Effect on the Treated (TOT)—the impact of the intervention on those who were actually “treated”—we would have to assume an absence of spillovers from participants to non-participants within a given *kebele*. Given the nature of how information is shared between peers within a *kebele*, we argue that this assumption is overly restrictive. The other reason is operational. From a policy perspective, ITT estimates are often the more relevant estimates because they measure average changes in outcomes across all individuals that are targeted by the intervention. Given that 100 percent compliance with the intervention is nearly impossible in a real-world scenario, ITT estimates are a good proxy for impacts that can be expected beyond the experimental study setting.

We estimate ITT effects using the following specification:

(1) $$y_{i}=\alpha +\beta T_{k}+X_{i}^{\prime }\delta +\mu_{w}+\varepsilon_{i}$$

where $y_{i}$ denotes the level of outcome y measured at the household level i (for instance whether the household has tried row-planting over the study period). The variable $T_{k}$ indicates the treatment status of *kebele* k which, in this specification, clubs both treatment arms (“Video” and “Video + Spouse”) into a single treatment (“Treatment”). The variable X is a vector of household- and development group-level characteristics that account for imbalances between groups. These include distance to nearest FTC, whether the household head received a formal education, distance to nearest dry season road, distance to nearest all-weather road, distance to nearest marketplace, and distance to DA office/house. We account for *woreda*-level stratification of our design through $\mu_{w}$, a set of *woreda*-level fixed effects. Lastly, we account for treatment assignment at the *kebele* level by clustering standard errors at that level.

We also estimate ITT effects for each of the two treatment arms that measure the differential impact of video-mediated extension when it is targeted only to heads of households (“Video”) and when it includes both the heads and spouses in the same household (“Video + Spouse”). This differential effect is estimated as:

(2) $$y_{i}=\alpha +\beta^{1}T_{k}^{1}+\beta^{2}T_{k}^{2}+X_{i}^{\prime }\delta +\mu_{w}+\varepsilon_{i}$$

where $T_{k}^{1}$ is treatment for “Video” and $T_{k}^{2}$ is treatment for “Video + spouse”. We also test for the equality of coefficients between “Video” and “Video + spouse” (i.e., $\beta^{1}=\beta^{2}$) to assess the additional effect of treating spouses in households where the head of the household is treated.

As described above, DAs in treatment groups were encouraged (but not compelled, and not monitored) to first focus their video-mediated extension efforts on 10 development groups from which we later sampled households randomly for our household survey. This design may lead to an over-representation of extension participants in our treatment groups as compared to the control group. Further, if DAs in the control group targeted their effort to particular types of development groups (for instance, those closer to FTCs), extension participants may not be fully comparable across samples. While our main strategy relies on the ITT estimations, we also test for the robustness of these results by restricting the sample to those development groups effectively reached by DAs in the treatment *kebeles*, that is, those development groups where at least one farmer participated in the video-mediated extension approach (Appendix F).

## 6 Results and discussions

### 6.1 Technology uptake

Results point to a clear and meaningful effect of the video-mediated extension approach on technology uptake. Specifically, the results show a 6 percentage points increase in uptake in year 1 and a 7 percentage point increase in year 2, which translate into 10 percent and 11 percent increases in the mean uptake rate compared to the control group (Table 4). As discussed earlier, differences between the two years are essentially driven by relatively more treatment farmers trialing a technology in the first year and continuing in the second year. In other words, the results show sustained uptake of technologies (i.e., adoption in both years) by treatment households (Appendix D).

**Table 4:** Estimates of treatment effects on technology adoption, by technology and year.

	Uptake of technology							
	Any technology		Row planting		Precise seeding rate		Urea top/side dressing	
*Panel A: meher 2017/18 (year 1)*								
Pooled treatment	0.0624***		0.0558***		0.0831***		0.0748***	

(T1 + T2)	(0.0187)		(0.0204)		(0.0218)		(0.0202)	

Video treatment		0.0787***		0.0657***		0.0982***		0.0801***
(T1)		(0.0226)		(0.0244)		(0.0260)		(0.0232)

Video + Spouse treatment		0.0470**		0.0464**		0.0690***		0.0698***
(T2)		(0.0208)		(0.0227)		(0.0250)		(0.0235)

F Test		2.056		0.680		1.218		0.192
Prob F		0.153		0.410		0.271		0.662
Constant	0.651***	0.651***	0.457***	0.457***	0.448***	0.449***	0.469***	0.470***
	(0.0256)	(0.0256)	(0.0261)	(0.0261)	(0.0300)	(0.0299)	(0.0268)	(0.0267)
Control mean	0.636	0.636	0.428	0.428	0.405	0.405	0.485	0.485
Observations	2,345	2,345	2,345	2,345	2,345	2,345	2,345	2,345
R-squared	0.285	0.286	0.429	0.429	0.168	0.169	0.313	0.313

*Panel B: meher 2018/19 (year 2)*								
Pooled treatment	0.0739***		0.0714***		0.0725***		0.0405	
(T1 + T2)	(0.0249)		(0.0244)		(0.0253)		(0.0257)	

Video treatment		0.0816***		0.0824***		0.0824***		0.0424
(T1)		(0.0287)		(0.0279)		(0.0285)		(0.0299)

Video + Spouse treatment		0.0666**		0.0610**		0.0630**		0.0386
(T2)		(0.0274)		(0.0284)		(0.0283)		(0.0290)

F Test		0.346		0.579		0.564		0.0169
Prob F		0.557		0.447		0.453		0.897
Constant	0.686***	0.686***	0.431***	0.431***	0.459***	0.460***	0.507***	0.507***
	(0.0299)	(0.0299)	(0.0286)	(0.0286)	(0.0333)	(0.0333)	(0.0313)	(0.0314)
Control mean	0.664	0.664	0.440	0.440	0.441	0.441	0.507	0.507
Observations	2,119	2,119	2,119	2,119	2,119	2,119	2,119	2,119
R-squared	0.170	0.170	0.353	0.353	0.222	0.222	0.225	0.225

*Note*: Robust standard errors in parentheses, clustered at the *kebele* level. *Woreda* fixed effects. Controls for distance to nearest FTC (categories), whether household head received formal education, distance to nearest dry season road, distance to nearest all-weather road, distance to nearest marketplace, and distance to DA office/house. *** p < 0.01, ** p < 0.05, * p < 0.

Disaggregating the results by technology, we find similar patterns. Overall, the video-mediated approach results in a 13 percent, 20 percent and 15 percent increase in uptake over control group means in year 1 for row planting, precise seeding rate, and urea top/side dressing, respectively. Similar results are found in year 2 for the first two technologies, even despite evidence of large increases in uptake in the control group.^16^ Crop-specific estimates vary but are generally positive and statistically significant across both years for most crops (Appendix E). The heterogeneous results by crop are explained by the fact that the novelty of the promoted practices varies by crop (e.g., row planting is new for teff and wheat but not for maize production). In neither year do we find the effects to be statistically different in magnitude across T1 and T2 (note the insignificant F statistics in column 2). This suggests that the gendered treatment—the addition of (typically female) spouses to the video-mediated approach—did not have an incremental effect on uptake. We explore this finding later in the paper.

### 6.2 Pathways and mechanisms

In unpacking these results, we examine a set of intermediate outcomes to explain the effect of video-mediated extension on farmers’ uptake of the technologies described above.

In Table 5 we present results for the effect of the video-mediated approach on farmers’ access to agricultural extension. At the extensive margin (columns 1–2), farmers within treatment *kebeles* were 17 percentage points more likely to have had direct contact with a DA in the first year (through extension sessions or field visits) than those in the control group (a 40 percent increase over the control mean). Results hold when combined with the intensive margins (columns 3–4), with close to 40 percent more trainings attended by farmers in the treatment groups. Results hold, albeit with a lower magnitude in year 2. They also hold in both treatment groups, with no statistically significant difference between them, or across crops (Appendix G).

**Table 5:** Estimates of treatment effects on extension access (for the household head), any technology.

	Extension access for household head, any technology							
	DA provided advice/training		No. of times DA provided advice/training		DA visited plot		No. of plots visited by DA	
*Panel A: meher 2017/18 (year 1)*								
Pooled treatment	0.169***		0.967***		0.0622***		0.155***	
(T1 + T2)	(0.0203)		(0.169)		(0.0223)		(0.0514)	
Video treatment		0.167***		1.080***		0.0690***		0.171***
(T1)		(0.0242)		(0.213)		(0.0266)		(0.0616)
Video + Spouse treatment		0.171***		0.860***		0.0558**		0.139**
(T2)		(0.0232)		(0.186)		(0.0244)		(0.0584)
F Test		0.0229		1.074		0.282		0.262
Prob F		0.880		0.301		0.596		0.609
Constant	0.524***	0.524***	2.408***	2.414***	0.382***	0.383***	0.769***	0.770***
	(0.0281)	(0.0282)	(0.207)	(0.207)	(0.0292)	(0.0292)	(0.0694)	(0.0694)
Control mean	0.472	0.472	2.306	2.306	0.340	0.340	0.676	0.676
Observations	2,345	2,345	2,345	2,345	2,345	2,345	2,344	2,344
R-squared	0.324	0.324	0.289	0.289	0.261	0.261	0.316	0.316

*Panel B: meher 2018/19 (year 2)*								
Pooled treatment	0.0814***		0.462***		0.0281		0.0223	
(T1 + T2)	(0.0263)		(0.115)		(0.0210)		(0.0417)	
Video treatment		0.0787**		0.465***		0.0221		0.00126
(T1)		(0.0307)		(0.138)		(0.0244)		(0.0499)
Video + Spouse treatment		0.0840***		0.459***		0.0339		0.0422
(T2)		(0.0295)		(0.134)		(0.0240)		(0.0472)
F Test		0.0335		0.00202		0.247		0.683
Prob F		0.855		0.964		0.620		0.409
Constant	0.488***	0.488***	1.499***	1.499***	0.296***	0.296***	0.566***	0.565***
	(0.0315)	(0.0315)	(0.169)	(0.170)	(0.0273)	(0.0273)	(0.0604)	(0.0604)
Control mean	0.426	0.426	1.297	1.297	0.246	0.246	0.446	0.446
Observations	2,119	2,119	2,119	2,119	2,119	2,119	2,116	2,116
R-squared	0.191	0.191	0.168	0.168	0.219	0.219	0.243	0.243

*Note*: Robust standard errors in parentheses, clustered at the *kebele* level. *Woreda* fixed effects. Controls for distance to nearest FTC (categories), whether household head received formal education, distance to nearest dry season road, distance to nearest all-weather road, distance to nearest marketplace, and distance to DA office/house. *** p < 0.01, ** p < 0.05, * p < 0.1.

Columns 5–8 focus on DA-level efforts to visit farmers’ field, that is, outside the group-level extension where main extension messages are conveyed. These visits allow for the provision of advice specific to a farmer’s plots, and to follow-up on messages provided during the group extension sessions. Our results point to an 18 percent increased probability of field visits on farmers’ plots in the treatment group as compared to the control group in year 1, with no distinction across the two treatment groups. Because these visits do not encompass video mediation, we interpret these results as indicators of DAs’ own efforts, which may be affected by their sense of effectiveness raised by the video-mediation approach. The results however do not persist in year 2, either because of a lesser need for extension support among farmers in treatment groups who gained experience from trialing in year 1, and/or because of DAs’ need for effort reallocation to other development groups, and/or to a lowering of their momentary increase in motivation.

Although the study design does not allow us to separately test for the effect of video-mediated extension on farmers’ (DAs’) motivation to seek out (provide) agricultural extension, we find evidence for both mechanisms in year 1. While the treatment effect on farmers’ attendance in extension sessions persists in year 2, we no longer find evidence of DAs’ increased efforts beyond leading collective extension sessions. Whether this lack of evidence is indicative of a momentary increase in DAs’ motivation in year 1 only, or a reallocation of efforts in year 2 towards other development groups is a key question as it affects the sustainability of impacts over time.

Next, we assess whether access to video-mediated extension translated into increased knowledge of the subject technologies (Table 6), either from better, more consistent messaging or from higher participation in extension sessions, as just described. Using percentage score to measure knowledge, we find that farmers in treated *kebeles* perform better than respondents in control *kebeles* in year 1 (a 3 percent increase for both household heads and spouses). However, these positive knowledge effects of the video-mediated approach dissipate in year 2. The likely explanation for this—seen in the difference between the control means in years 1 and 2—is that the control group “caught up” with the treatment group. As before, these results hold across both treatment groups for (typically male) household heads.

**Table 6:** Estimates for treatment effects on knowledge of subject technologies.

	Knowledge score, percentage			
	Head of household		Spouse	
*Panel A: meher 2017/18 (year 1)*				
Pooled treatment	1.354**		1.141*	
(T1 + T2)	(0.529)		(0.666)	

Video treatment		1.103*		0.996
(T1)		(0.615)		(0.775)

Video + Spouse treatment		1.591**		1.279*
(T2)		(0.619)		(0.749)

F Test		0.591		0.146
Prob F		0.443		0.702
Constant	40.48***	40.47***	36.62***	36.61***
	(0.679)	(0.680)	(0.877)	(0.876)
Control mean	39.93	39.93	35.31	35.31
Observations	2,345	2,345	1,839	1,839
R-squared	0.179	0.180	0.230	0.230

*Panel B: meher 2018/19 (year 2)*				

Pooled treatment	0.150		1.267*	
(T1 + T2)	(0.517)		(0.726)	

Video treatment		0.409		1.207
(T1)		(0.596)		(0.814)

Video + Spouse treatment		−0.0953		1.323
(T2)		(0.585)		(0.834)

F Test		0.778		0.0221
Prob F		0.378		0.882
Constant	45.34***	45.36***	36.21***	36.21***
	(0.729)	(0.730)	(0.919)	(0.919)
Control mean	45.62	45.62	34.69	34.69
Observations	2,119	2,119	1,686	1,686
R-squared	0.128	0.128	0.223	0.223

*Note*: Robust standard errors in parentheses, clustered at the *kebele* level. *Woreda* fixed effects. Controls for distance to nearest FTC (categories), whether household head received formal education, distance to nearest dry season road, distance to nearest all-weather road, distance to nearest marketplace, and distance to DA office/house. *** p < 0.01, ** p < 0.05, * p < 0.1.

We also find significant and positive effects on (typically female) spouse’s access to extension based on responses to the questionnaire deployed to spouses in year 1, though not in year 2 (Appendix G). These effects are observed on both the extensive margin (access to advice or training from DAs) and the intensive margin (the number of times such advice or training was received from a DA). In columns 3–4, we further assess changes in knowledge using the spouse questionnaire. We find qualitatively similar results in year 1 that persist/continue into year 2 possibly due to limited increases in knowledge among spouses in the control group.

Taken together, the impact of the gendered treatment arm on extension access and knowledge outcomes for spouses indicate that the video-mediated extension approach resulted in significant gains at early points in the technical change pathway. The question is why higher-level outcomes—increases in uptake—are indistinguishable from the spouse-only treatment. One simple explanation is that decision-making on these crops and technologies is controlled by male household heads. Indeed, the gendered specialization of decision-making on labor and resource allocations within agricultural households is often used to explain why women are not targeted by extension services for certain types of technologies (e.g., production technologies for cereal crops) versus others (e.g., nutrition- and health-related technologies for other crops) (see Doss, 2015).

But this may be more heuristic than empirical: there is ample evidence to suggest that few technologies can be readily reduced and assigned as “male” or “female.” Rather, the evidence strongly points to gender biases in extension targeting (e.g., Doss and Morris, 2001, Palacios-López and López, 2015) coupled with complex intra-household decision-making processes (e.g., Udry, 1996, Doss and Morris, 2001, Hoel et al., 2021). As such, our findings are consistent with prior studies on Ethiopia that identify both gender biases in extension service provision and heterogeneity in intra-household gender relations in rural Ethiopia (Mogues et al., 2019, Ragasa et al., 2013, Buchy and Basaznew, 2005 in Ethiopia). Moreover, as indicated in Section 3, spouses were exposed to similar videos shown to household heads (typically male) and the videos could be less salient or appealing to women, and thus generated no additional impacts.

### 6.3 Yield effects

Our results for yields (Table 7) are slightly less encouraging than those obtained for technology uptake, although not surprising. Using our self-reported household yield measurements, we find a slightly significant and positive impact of the video-mediated approach on teff yields in year 1, but no significant impact on wheat and maize yields. We also find that the effect on teff yields effectively disappears in year 2. These results are robust to our other measurements of yield: self-reported plot-level yield measurements do not offer additional evidence of significant increases resulting from the treatment, apart from a slightly significant and positive impact on teff yields when plot area is measured with GPS devices (Appendix E). And again, we observe no differences between the standard video-mediated treatment and the gendered version of the treatment.

**Table 7:** Estimates of treatment effects on yield, by crop.

	Self-reported yield, household level					
	Teff		Wheat		Maize	
*Panel A: meher 2017/18 (year 1)*						
Pooled treatment	0.653*		0.178		1.019	
(T1 + T2)	(0.388)		(0.778)		(1.134)	

Video treatment		0.807*		−0.125		1.619
(T1)		(0.479)		(0.964)		(1.221)

Video + Spouse treatment		0.498		0.462		0.441
(T2)		(0.421)		(0.890)		(1.286)

F Test		0.450		0.340		1.185
Prob F		0.503		0.560		0.277
Constant	8.698***	8.701***	17.85***	17.84***	24.83***	24.86***
	(0.500)	(0.501)	(0.821)	(0.822)	(1.282)	(1.287)
Control mean	8.447	8.447	18.74	18.74	23.82	23.82
Observations	1,498	1,498	1,442	1,442	1,301	1,301
R-squared	0.188	0.189	0.231	0.232	0.295	0.295

*Panel B: meher 2018/19 (year 2)*						
Pooled treatment	0.263		−0.0432		1.116	
(T1 + T2)	(0.422)		(0.675)		(1.070)	

Video treatment		0.474		0.283		2.037*
(T1)		(0.464)		(0.858)		(1.194)

Video + Spouse treatment		0.0473		−0.371		0.255
(T2)		(0.501)		(0.707)		(1.245)

F Test		0.823		0.658		2.284
Prob F		0.365		0.418		0.132
Constant	8.110***	8.129***	17.38***	17.39***	23.78***	23.89***
	(0.476)	(0.477)	(0.883)	(0.884)	(1.372)	(1.377)
Control mean	8.280	8.280	18.09	18.09	23.80	23.80
Observations	1,256	1,256	1,304	1,304	1,042	1,042
R-squared	0.278	0.278	0.396	0.396	0.334	0.335

*Note*: Robust standard errors in parentheses, clustered at the *kebele* level. *Woreda* fixed effects. Upper end of the yield distribution winsorized at the 1 percent level. Controls for distance to nearest FTC (categories), whether household head received formal education, distance to nearest dry season road, distance to nearest all-weather road, distance to nearest marketplace, and distance to DA office/house. *** p < 0.01, ** p < 0.05, * p < 0.1.

Potential measurement issues and the subject technologies themselves—many of which have a history of mediocre performance despite being flagships of the government’s efforts to increase yields and improve food security—may jointly contribute to these results. For example, reporting results from a randomized controlled trial of the government’s wheat technology package—comprised of practices similar to those investigated in our study—Abate et al. (2018) found a 14 percent increase on wheat yields, but only when measured with crop cuts and when using farmer predicted (rather than farmer self-reported) yields. In fact, they found that farmers’ actual reported yields showed smaller and statistically insignificant yield gains attributable to the package. They attribute this difference in outcomes to measurement errors in plot size and advise caution in the use of both farmer-reported plot sizes and output. This is consistent with results from a similarly experimental study of teff row planting and lower seeding rates conducted by Vandercasteelen et al., 2020, Vandercasteelen et al., 2018. They found no statistically significant effects of the teff package on yields, whether measured with data from crop cuts or calculated from farmer-reported output and enumerator-measured area based on GPS data.

### 6.4 Robustness checks

Finally, we test for the robustness of main results (i.e., technology uptake, mechanisms, and yield effects) by restricting the sample to those development groups effectively reached by DAs in the treatment or control *kebeles*, that is, those development groups where at least one farmer participated in the video-mediated extension approach. A description of the sample size, uptake trends, and main results are provided in Appendix F. Our results are not meaningfully affected by this restriction of our sample and remain consistent with uptake estimates reported above and our estimates of pathways, mechanisms and yields. As such, the ITT estimation results obtained are unlikely to be driven by selection bias and can be interpreted as credible estimates of the video-mediated extension approach’s impact.

## 7 Cost implications

Because the video-mediated approach is designed to augment the existing extension system, we investigate the cost per additional uptake (or “adoption”, for convenience in exposition) resulting from the video-mediated approach. Specifically, we measure marginal cost-effectiveness, which is the cost of an additional adoption that results from adding the video-mediated extension approach to the existing system (see the methods we follow to estimate marginal cost-effectiveness in Appendix H).

We examine two scenarios: (i) the actual cost of Digital Green’s project per adoption attributable to the video-mediated approach (our experimental scenario), and (ii) the estimated cost of a larger-scale rollout that covers the target *kebeles* and *woredas* more completely (our saturation scenario). Overall, we find that while the cost of an additional adoption of a subject technology under the experimental scenario ranges from USD $16–30, it significantly drops to USD $3–6 in the saturation scenario. Given that so few cost-effectiveness analyses of extension initiatives have been undertaken to complement randomized evaluations such as ours (see Mogues et al. (2019) for an exception), it is difficult to determine how these marginal values compare. However, we would suggest that at a saturation scale, the program is potentially cost effective.

## 8 Discussion

Several important findings emerge from our evaluation of the video-mediated extension approach employed by the MoA, Digital Green, and the regional bureaus of agriculture across Ethiopia’s main agricultural regions. *First*, the approach demonstrates its capacity to reach a wider audience than the conventional approach employed by DAs and *woreda*-level extension staff. More importantly, we found that the video-mediated approach increases extension reach even when we restrict our sample to those development groups where at least one farmer received advice from a DA, indicating that the approach did not lead to a change in the type of development groups that DAs decided to work with, but rather to a change in their reach to farmers within these groups. One plausible explanation for increased extension reach from the qualitative research conducted as part of this study is that video screenings tend to enhance DAs’ capacities to organize farmers at a given location and time, partly due to increased interest by farmers in the medium.

*Second*, the approach leads to higher levels of knowledge about the subject technologies in the first year, with gains observed for both household heads and spouses. This effect on knowledge can be explained by the power of video for delivering technical content in a consistent manner, thereby reducing oversights in conveying information that requires more accuracy than an extension agent may be able to retain/remember and communicate correctly. Moreover, the visual aspect (being able to see how a given technology or practice is implemented first-hand) was indicated by farmers as an important element of the approach in terms of maximizing learning (technical understanding).

*Third*, the video-mediated approach results in increased uptake of the technologies that are central to the extension program of the MoA and regional bureaus, which endure in the second year of the experiment, indicating farmers’ effective adoption of technologies beyond a mere trial in one production season. While increased extension reach and knowledge due to the video-mediated approach are the main mechanisms that explain the sustained technology uptake, the qualitative evidence specifically identifies the local character (farmer) featured in the videos as a key aspect of the approach that encourages farmers to adopt promoted technologies. This is consistent with several theoretical and empirical studies that demonstrate the effects of role models who are similar to individuals across multiple dimensions in facilitating learning that can lead to desirable changes in behavior (e.g., Bernard et al., 2014, Bandura, 1977, Bandura, 1986).

While these results hold when both the head of household and the spouse are targeted by the video-mediated approach, we do not observe any marginal gains in uptake rates by treating both. In other words, while more spouses had access to extension and they have tended to learn more from it under the “Video + Spouse” treatment arm, this did not translate in changes in households’ technology adoption decisions above and beyond that of the “Video” treatment arm. This result implies that efforts to improve women’s role in technology adoption decision should go beyond attempting to create equal access to extension services, but also address underlying inequalities in intra-household bargaining power. Regarding the topic at hand, this clearly suggests the need for further analysis of the gender dimensions of video-mediated extension. For instance, there is a value in disentangling inequalities in intra-household bargaining power from social institutions and cultural norms, and further disentangling these effects from gender-unintentional practices within the extension system.

Although our study is primarily designed to assess the effect of video-mediated extension on adoption, we also investigate the resulting effects of adoption on farmers’ yields. However, we do not find statistically significant yield effects resulting from the adoption of promoted practices. We attribute this to challenges in accurately measuring both output and area, as well as the possibility that the technological package being promoted has a limited effect on productivity. We used farmer self-reported area and production to estimate yield and we recognize that such data often lead to measurement errors; and recent evidence from Ethiopia demonstrates the extent and magnitude of this problem vividly (Abate et al., 2018, Abay et al., 2019). This opens the door for future research that integrates more accurate ground-truthing methods for yield measurement such as crop-cuts with yield estimation using satellite imagery and associated analytical tools.

Despite this, it is also important to recognize the wider policy relevance of our findings. Unlike many prior studies on ICTs in agricultural extension, the program studied here represents a large-scale intervention of the Government of Ethiopia that is fully integrated into existing policy and practice. In other words, we do not seek to assess the effect of a new agricultural extension approach to *replace* existing ones, but instead focus on the effect of *complementing* existing systems with video. Our results provide evidence of the potential contribution of adding video mediation to existing extension policy and programming in Ethiopia and encourage further innovation in the program’s design to generate improved outcomes.

Because video is an added feature to the regular extension system, we further assess the *marginal* cost-effectiveness of adding the video-mediated approach to existing extension, under the experimental and a full saturation scenario. The cost of each additional adoption under the experimental scenario ranges from USD 16 – 30. However, these figures decrease to USD 3 – 6 when we assume that the video-mediated approach is extended to all *kebeles* in the treatment *woredas*. The approach’s cost-effectiveness will further increase as the number of woredas (and thereby viewers) increases, given that the video production costs are fixed costs. It is important to emphasize that these figures relate to the cost of increasing adoption beyond the levels obtained by the regular extension system.

Overall, this study helps shift focus in national and global discourse on agricultural extension to the power that ICTs can have in augmenting—rather than replacing—extension services and agents through the development and dissemination of targeted/site-specific advisories in a flexible and effective manner. This, in turn, may draw attention to more constructive ways of thinking about lowering costs, improving efficiency, and increasing the impact of existing extension systems. As Ethiopia and other low-income countries explore innovative ways to strengthen their extension and advisory services to farmers, these findings provide evidence on what works—and for whom—in the arena of innovative extension methods and tools.

## 9 Conclusions

This study assess whether video-mediated agricultural extension leads to increased and sustained uptake of agricultural technologies and practices by smallholder farmers. We explore this question in the context of a large-scale experimental rollout of a video-mediated extension approach by the Government of Ethiopia and Digital Green over a two-year period (the main production seasons in 2017/18 and 2018/19) in 347 kebeles across the four main regions. Based on a sample of 2345 households, we find that video-mediated extension approach led to increases in the uptake of key agricultural technologies and practices by farmers. Specifically, we find a 6-percentage point overall increase in farmers’ uptake of the recommended technologies in the first year of the experiment, which translates into a 10 percent increase over the mean of the control group. Across technologies, we find that the video-mediated approach resulted in a 13, 20 and 15 percent increase in uptake over control group means for row planting, precise seeding rate, and urea top/side dressing, respectively. These results endure in the second year of the experiment, indicating farmers’ effective adoption of the technologies beyond a mere trial in one production season.

We also study whether video-mediated extension targeted at both spouses of a household is more effective than when only targeted at the (typically male) household head. While the results point to greater participation and greater agricultural knowledge of spouses who also received the video-mediated extension, we do not find clear evidence that targeting both spouses translated into higher uptake of technologies. Likewise, we do not find clear evidence that the increased adoption of the technologies promoted by the Ethiopian public extension system translated into higher yields. These null yield results may be related to issues of measurement error, insufficient adaptation of the technologies to context, limited investment in complementary inputs or other factors.

Looking at the mechanisms that explain the effects of the approach on adoption of technologies, we find that the video-mediated extension approach led to an increase in extension reach, with a 35 percent increase in farmers’ attendance at extension sessions, likely due to increased interest by farmers in the medium. In turn, we find a higher level of knowledge—greater technical understanding of the subject technologies and practices—among farmers in kebeles randomly assigned to the video-mediated extension approach.

However, these results come with one caveat: as for any new intervention, we are at this stage unable to assess whether our results are driven by the main features of the Digital Green approach, or by its novelty which may partly drive the observed higher participation of farmers to extension groups, and higher dissemination efforts by DAs. While our study covers two consecutive agricultural seasons, the long-term effect of such interventions remain open to further studies.

## Declaration of Competing Interest

The authors declare that they have no known competing financial interests or personal relationships that could have appeared to influence the work reported in this paper.

## Data Availability

The data is available in the IFPRI Dataverse.

## Footnotes

1 For the sake of brevity, we use the term “extension” hereafter to agricultural extension and advisory services.

2 This estimate is based on the CSA (2016) estimate of more than 17 million farm households who are active in agriculture at this time. See Davis et al. (2010) for comparison figures from other countries.

3 A *woreda* in Ethiopia is analogous to a district or county in other countries, while *kebeles* are village clusters that constitute the lowest administrative unit in Ethiopia.

4 For brevity, we refer to these suites as “technologies” in the remainder of this paper. Note, however, that the suites being promoted by the video-mediated approach are, in fact, a complex set of principles, practices, technologies, and inputs that are accompanied by a set of precise decision rules that farmers need to follow in order to successfully apply them (Appendix A). See Abate et al. (2018) and Vandercasteelen et al., 2020, Vandercasteelen et al., 2018 for crop-specific examples directly relevant to this study, and Stevenson et al. (2019) for a broader discussion.

5 The four regional states are: Amhara; Oromia; Southern Nations, Nationalities, and Peoples (SNNP); and Tigray.

6 The current extension approach is described as the Participatory Extension System (PES), highlighted by the organization of farmers in development groups and social networks such as the “one-to-five” syndicates to share information. However, there is little to suggest that these approaches innovate on the traditional top-down training methods implicit in previous approaches.

9 The public extension bureau in each study *woreda* produced and disseminated at least three types of videos on each of the three focus crops covering the non-negotiable practices (i.e., row planting, seeding rate, and fertilizer application).

10 Participation and adoption data are filled out by DAs, both in the treatment and control *kebeles*, except that DAs in the treatment *kebele* use the COCO platform and DAs in control *kebele* use a paper-based process. Thus, we estimate no significant difference in the intensity of engagement between DAs and farmers across *kebeles* due to the type of the monitoring tool.

11 To the greatest extent possible, our design sought not to affect the way extension was conducted in the control *kebeles*. For this reason, we did not encourage DAs to focus their attention on 10 development groups as we did in the treatment *kebeles*.

12 We conducted a mean difference test between attritors (n=77) and non-attritors (n=2,345) and found statistically insignificant difference on the vast majority of household-, location-, and plot/production-related characteristics. Moreover, there is no statistically significant correlation between attrition status and treatment status.

13 Our qualitative observation indicates that videos were not screened in the remainder 15 (17) percent of the *kebeles* in year 1 (year 2) mainly due to technical issues (e.g., functionality of the PICO projectors) and turnover of DAs trained on video screening.

14 We also use a weighted knowledge test score along the lines of Shikuku (2019) where the inverse of the probability of answering a question correctly is used to weight correct responses.

15 A feasibility study was conducted to estimate the cost of conducting crop cuts to provide a more definitive measure of yield. However, it was determined not only to be prohibitively costly to implement, but also logistically challenging to ensure that crop cuts were taken in timely manner for each crop: asynchronous planting dates at the *kebele* level, variation in crop duration, and the vagaries of weather were all key factors.

16 We interpret this increase in uptake in the control group as evidence that efforts to promote the subject technologies using the conventional extension approach were also successful to a measurable degree.

## Footnotes

7 In our sample, 90 percent of households are male headed (see Appendix Table C1).

8 Similar videos were screened for both household head and his/her spouse in separate sessions. In other words, the videos screened to spouses (typically female) were not tailored in any way to specifically appeal to women. It is also worth mentioning that spouses are not usually targeted by the conventional agricultural extension approach.
