# Supplementary material for: Accelerating technical change through ICT: Evidence from a video-mediated extension experiment in Ethiopia
Source: World Dev. 2023 Jan;161:106089. doi: 10.1016/j.worlddev.2022.106089 (PMC9693707; doi:10.1016/j.worlddev.2022.106089)
Supplement: Supplementary data 1 [file mmc1.docx]

**Online appendix**

**Accelerating technical change through ICT: Evidence from a video-mediated extension experiment in Ethiopia**

# Appendix A: Subject technologies and practices promoted through the video-mediated extension

This study focuses on three suites of technologies and practices that were promoted through the video-mediated extension approach. Several of these technologies have figured prominently in past efforts to accelerate productivity growth in cereal staples by the extension system, and by organizations such as Sasakawa Global 2000, a non-governmental organization that piloted the PADETES approach with the MoA during the 1990s. Others are more recent entrants into the landscape, gaining attention through the Ethiopian Agricultural Transformation Agency (ATA), which has actively promoted packages comprising these three practices (ATA, 2014). These include teff, wheat, and maize row planting, reduced (precise) seeding rate, and urea top/side dressing. We therefore expect to find differential effects of the intervention across the three crops. Details of these technologies are as follows.

*Row planting*—planting in row with spacing between plants and rows—is a recommended agronomic practice to ensure proper light interception, which is among the main factors that determine crop growth (Charles-Edwards, 1982). It also facilitates better weed management and ensures even distribution of seeds and thereby uniform access for plan inputs like water and nutrients (Fufa et al., 2011). Row planting has been shown to increase the number of plant tillers, number of kernels per spikes, and seed weight which contributes to increase in yields under research station trials (Berhe et al., 2011; Fufa et al., 2011; Lafond, 1994). However, recent farmer level experimental evidence on row planting for teff and wheat suggest much lower impact (Vandercasteelen et al., 2018; Abate et al., 2018). For maize, row planting has been promoted in Ethiopia for several decades and is a more established practice. Despite this, broadcasting seeds is not an uncommon practice among Ethiopia’s maize farmers.

*More precise seeding rates* have been shown to increase yield by ensuring even distribution of seed and thereby reducing the competition between plants for water, light, and nutrients (Fufa et al., 2011). Reducing seed rate also allows for optimal trilling and increases the number of kernels per spike (Carr et al., 2003). More precise seeding rates are often associated with row planting: for teff and wheat, row planting requires lower seeding rates, while for maize it may actually require a higher seeding rate depending on how the farmer otherwise broadcasts maize seed. However, lower seeding rates for teff and wheat is also recommended for those farmers that broadcast plant.

*Urea top or side dressing* is another recommended practice associated with the strategy of multiple nitrogen applications, i.e., splitting nitrogen application to make efficient use of nitrogen fertilizer when it is difficult to precisely assess the mineral nitrogen content of the soil and predict the nitrogen requirement of the crop *ex ante* (Spiertz and De Vos, 1983; Baligar and Bennett, 1986). Top dressing or foliar urea fertilization, in particular, is promoted because it reduces potential nitrogen losses and has the ability to provide nitrogen when root activities are impaired, for instance, under dry conditions (Gooding and Davies, 1992). While row planting and more precise seeding rates are relatively novel practices for many Ethiopian teff and wheat farmers, they are more commonly practiced—though not universally—by Ethiopian maize farmer.

# Appendix B: Study timeline

**Table B1. Intervention and study timeline**

| Year | **Year 1** | | | | | | **Year 2** | | | | | |
| --- | --- | --- | --- | --- | --- | --- | --- | --- | --- | --- | --- | --- |
|  |  | 2017 | | 2018 | | | 2018 | | 2019 | | | |
| Month | Jan-Mar | Apr-May | Jun-Dec | Jan | Feb | Mar | Apr-May | Jun-Dec | Jan | Feb | Mar | Jun-Aug |
| Activity | Qualitative survey | DA training on video-based extension  DA survey | Video-mediated extension provision  Adoption verification survey | Household and DA survey | | | DA Refresher training on video-based extension | Video-mediated extension provision  Adoption verification survey | Household and DA survey | | | Qualitative survey |
| Season | Marketing  season | Marketing season | Main production and harvesting season | Marketing season | | | Marketing season | Main production and harvesting season | Marketing  season | | | Marketing  season |

# Appendix C: Experimental integrity (baseline balance and compliance)

We assess the extent to which random assignment of the treatment generated comparable treatment and control groups at different levels—*kebele*, household head, and spouse. First, we check for balance between treatment and control groups for the pooled treatment and find that these groups are comparable on household level variables (Table C1). Second, we check for balance across treatment arms and the results indicate the control and treatment groups are comparable on household characteristics, remoteness, and cropping pattern (Tables C2a and C2b).

**Table C2. Balance test for household level covariates, pooled treatment groups**

| Variable | Entire sample | Treated | Control | Treated − Control |
| --- | --- | --- | --- | --- |
| HH size | 5.945 (2.183) | 5.969 (2.191) | 5.896 (2.167) | 0.073 (0.126) |
| Male HH head | 0.901 (0.299) | 0.900 (0.300) | 0.903 (0.296) | -0.003 (0.014) |
| Age of HH head | 45.887 (12.932) | 45.975 (12.935) | 45.716 (12.934) | 0.259 (0.658) |
| HH head formal educ | 0.495 (0.500) | 0.471 (0.499) | 0.543 (0.498) | -0.072** (0.031) |
|  |  |  |  |  |
| Distance to the nearest (in minutes): | | | | |
| Asphalt road | 102.750 (84.914) | 104.407 (83.408) | 99.501 (87.755) | 4.906 (7.657) |
| Dry season road | 27.129 (41.147) | 29.294 (44.048) | 22.884 (34.395) | 6.410** (2.841) |
| All weather road | 30.498 (41.910) | 32.467 (43.519) | 26.636 (38.294) | 5.830** (2.908) |
| Market | 69.564 (56.420) | 72.072 (56.489) | 64.645 (55.994) | 7.427* (4.469) |
| Admin. center | 119.689 (85.980) | 122.749 (86.215) | 113.690 (85.251) | 9.059 (7.288) |
| Agri. coop | 49.601 (51.787) | 50.828 (52.889) | 47.197 (49.500) | 3.631 (3.740) |
| Input dealer | 56.969 (55.313) | 57.919 (53.514) | 55.106 (58.673) | 2.813 (4.381) |
| FTC | 31.997 (30.685) | 32.580 (30.686) | 30.854 (30.669) | 1.726 (1.806) |
| DA house/office | 32.741 (31.179) | 33.856 (32.456) | 30.554 (28.401) | 3.302* (1.875) |
| RuSACCOs^a^ | 78.238 (73.581) | 79.140 (71.697) | 76.470 (77.158) | 2.670 (6.617) |
| Microfinance | 103.216 (80.944) | 104.700 (79.840) | 100.306 (83.041) | 4.395 (7.012) |
| Bank | 115.491 (81.570) | 117.546 (80.724) | 111.462 (83.108) | 6.084 (7.180) |
|  |  |  |  |  |
| No. of parcels | 3.704 (2.154) | 3.684 (2.117) | 3.742 (2.224) | -0.058 (0.164) |
|  |  |  |  |  |
| In *meher* 2017/18: |  |  |  |  |
| HH cultivated teff | 0.639 (0.480) | 0.646 (0.478) | 0.624 (0.485) | 0.023 (0.042) |
| No. of teff plots | 1.075 (1.251) | 1.106 (1.289) | 1.016 (1.170) | 0.089 (0.102) |
| HH cultivated wheat | 0.615 (0.487) | 0.615 (0.487) | 0.615 (0.487) | 0.000 (0.042) |
| No. of wheat plots | 0.868 (0.933) | 0.844 (0.882) | 0.915 (1.024) | -0.071 (0.085) |
| HH cultivated maize | 0.555 (0.497) | 0.567 (0.496) | 0.530 (0.499) | 0.037 (0.044) |
| No. of maize plots | 0.702 (0.745) | 0.706 (0.717) | 0.694 (0.798) | 0.011 (0.071) |
| Observations (no.) | 2,345 | 1,553 | 792 | 2,345 |

*Note*: For columns 1-3, standard deviations in parentheses. For column 4, standard errors clustered at the *kebele* level in parentheses. *** p<0.01, ** p<0.05, * p<0.1. Outliers and missing observations replaced with *kebele* mean.

^a^ The term RuSACCO refers to a rural savings and credit cooperative.

**Table C3a. Balance test for household-level covariates, by treatment group**

| Variable | Entire sample | Video treatment  (T1) | Video + Spouse treatment (T2) | Control  (T0) | T1 - T0 | T2 - T0 | T2 - T1 | |
| --- | --- | --- | --- | --- | --- | --- | --- | --- |
| HH size | 5.945 | 6.007 | 5.933 | 5.896 | 0.110 | 0.036 | -0.074 | |
|  | (2.183) | (2.210) | (2.173) | (2.167) | (0.147) | (0.149) | (0.154) | |
| Male HH head | 0.901 | 0.895 | 0.905 | 0.903 | -0.007 | 0.002 | 0.010 | |
|  | (0.299) | (0.306) | (0.293) | (0.296) | (0.016) | (0.017) | (0.017) | |
| Age of HH head | 45.887 | 45.842 | 46.104 | 45.716 | 0.126 | 0.388 | 0.262 | |
|  | (12.932) | (12.964) | (12.913) | (12.934) | (0.748) | (0.747) | (0.707) | |
| HH head formal education | 0.495 | 0.458 | 0.483 | 0.543 | -0.085** | -0.060* | 0.025 | |
|  | (0.500) | (0.499) | (0.500) | (0.498) | (0.036) | (0.034) | (0.034) | |
| Distance to the nearest (in minutes): | | | | | | | |  |
| Asphalt road | 102.750 | 108.002 | 100.926 | 99.501 | 8.501 | 1.424 | -7.077 | |
|  | (84.914) | (78.851) | (87.503) | (87.755) | (8.467) | (9.016) | (8.467) | |
| Dry season road | 27.129 | 32.191 | 26.489 | 22.884 | 9.307** | 3.605 | -5.702 | |
|  | (41.147) | (47.408) | (40.363) | (34.395) | (3.700) | (3.143) | (3.854) | |
| All weather road | 30.498 | 36.194 | 28.858 | 26.636 | 9.557** | 2.222 | -7.336* | |
|  | (41.910) | (48.700) | (37.513) | (38.294) | (3.803) | (3.117) | (3.818) | |
| RuSACCOs^a^ | 78.238 | 80.677 | 77.651 | 76.470 | 4.207 | 1.182 | -3.025 | |
|  | (73.581) | (69.955) | (73.358) | (77.158) | (7.342) | (7.575) | (6.880) | |
| Microfinance | 103.216 | 106.516 | 102.943 | 100.306 | 6.210 | 2.637 | -3.573 | |
|  | (80.944) | (74.413) | (84.775) | (83.041) | (7.668) | (8.353) | (7.787) | |
| Bank | 115.491 | 120.595 | 114.594 | 111.462 | 9.133 | 3.132 | -6.000 | |
|  | (81.570) | (75.661) | (85.285) | (83.108) | (7.962) | (8.553) | (8.192) | |
| Observations (no.) | 2,345 | 764 | 789 | 792 | 1,556 | 1,581 | 1,553 | |

Note: For columns 1-4, standard deviations in parentheses. For columns 5-7, standard errors clustered at the *kebele* level in parentheses. *** p<0.01, ** p<0.05, * p<0.1. Outliers and missing observations replaced with *kebele* mean.

^a^ The term RuSACCO refers to a rural savings and credit cooperative.

**Table C2b. Balance test for household-level covariates on key farming characteristics, by treatment group**

| Variable | Entire sample | Video treatment (T1) | Video + Spouse treatment (T2) | Control  (T0) | T1 - T0 | T2 - T0 | T2 - T1 | |
| --- | --- | --- | --- | --- | --- | --- | --- | --- |
| No. of parcels | 3.704 | 3.683 | 3.686 | 3.742 | -0.059 | -0.057 | 0.002 | |
|  | (2.154) | (2.079) | (2.155) | (2.224) | (0.186) | (0.186) | (0.178) | |
| In *meher* 2017/18: |  |  |  |  |  |  |  | |
| HH cultivated teff | 0.639 | 0.665 | 0.629 | 0.624 | 0.041 | 0.005 | -0.036 | |
|  | (0.480) | (0.472) | (0.483) | (0.485) | (0.049) | (0.049) | (0.050) | |
| No. of teff plots | 1.075 | 1.098 | 1.113 | 1.016 | 0.082 | 0.096 | 0.015 | |
|  | (1.251) | (1.240) | (1.335) | (1.170) | (0.118) | (0.122) | (0.127) | |
| HH cultivated wheat | 0.615 | 0.606 | 0.624 | 0.615 | -0.009 | 0.009 | 0.018 | |
|  | (0.487) | (0.489) | (0.485) | (0.487) | (0.050) | (0.048) | (0.051) | |
| No. of wheat plots | 0.868 | 0.855 | 0.834 | 0.915 | -0.061 | -0.081 | -0.021 | |
|  | (0.933) | (0.933) | (0.830) | (1.024) | (0.099) | (0.092) | (0.088) | |
| HH cultivated maize | 0.555 | 0.577 | 0.558 | 0.530 | 0.047 | 0.027 | -0.020 | |
|  | (0.497) | (0.494) | (0.497) | (0.499) | (0.052) | (0.049) | (0.049) | |
| No. of maize plots | 0.702 | 0.721 | 0.691 | 0.694 | 0.027 | -0.004 | -0.030 | |
|  | (0.745) | (0.711) | (0.724) | (0.798) | (0.080) | (0.078) | (0.072) | |
| Distance to the nearest (in minutes): | | | | | | | |  |
| Market | 69.564 | 75.700 | 68.559 | 64.645 | 11.055** | 3.914 | -7.141 | |
|  | (56.420) | (58.234) | (54.554) | (55.994) | (5.318) | (5.095) | (5.353) | |
| Admin. center | 119.689 | 126.959 | 118.672 | 113.690 | 13.270 | 4.982 | -8.288 | |
|  | (85.980) | (83.274) | (88.834) | (85.251) | (8.170) | (8.735) | (8.599) | |
| Agri. coop | 49.601 | 51.297 | 50.373 | 47.197 | 4.101 | 3.176 | -0.924 | |
|  | (51.787) | (50.502) | (55.130) | (49.500) | (4.298) | (4.421) | (4.477) | |
| Input dealer | 56.969 | 61.399 | 54.549 | 55.106 | 6.293 | -0.557 | -6.850 | |
|  | (55.313) | (55.953) | (50.849) | (58.673) | (5.224) | (4.761) | (4.808) | |
| FTC | 31.997 | 32.981 | 32.192 | 30.854 | 2.127 | 1.338 | -0.789 | |
|  | (30.685) | (31.740) | (29.645) | (30.669) | (2.170) | (2.042) | (2.161) | |
| DA house/office | 32.741 | 35.086 | 32.665 | 30.554 | 4.532* | 2.111 | -2.421 | |
| Observations (no.) | 2,345 | 764 | 789 | 792 | 1,556 | 1,581 | 1,553 | |

Note: For columns 1-4, standard deviations in parentheses. For columns 5-7, standard errors clustered at the *kebele* level in parentheses. *** p<0.01, ** p<0.05, * p<0.1. Outliers and missing observations replaced with *kebele* mean.

We also measure experimental integrity using the incidence of treatment (i.e., compliance). We broadly define the term “incidence” as the share of units (development groups or *kebeles*) having participated in a video screening session or having received a visit from a DA. We use two different indicators to measure incidence, as follows.

- 1. *Development Group-level video screening.* This is an indicator variable that equals 1 if at least one sampled farmer belonging to a Development Group reported attending at least one video screening session on crop production, and 0 otherwise. In effect, this variable indicates whether a Development Group screened a video.
  2. *Kebele-level video screening*. This is an indicator variable that equals 1 if at least one sampled farmer living in a given *kebele* reported attending at least one video screening session on crop production, and 0 otherwise. In effect, this variable indicates whether at least one video was screened in a *kebele*.

Our descriptive analysis (Table C3) shows that video screenings took place in 59 percent (52 percent) of development groups in the sample in year 1 (year 2). We find similar results for both treatment arms. Extrapolated to a *kebele* level, videos were screened in 85 percent (83 percent) of *kebeles* in the pooled treatment group in year 1 (year 2). Also, worth noting is the very low rate of “contamination” across the experiment: in most cases, households in the control group did not report participating in the video-mediated extension approach. This is critical to maintaining the integrity of the experimental design.

**Table C4*.* Farmer-reported attendance at a video screenings**

| **Treatment group** | **Farmer-reported attendance at video screenings** | | | |
| --- | --- | --- | --- | --- |
|  | **Development group level** | | **Kebele level** | |
|  | **Year 1** | **Year 2** | **Year 1** | **Year 2** |
|  |  |  |  |  |
| Pooled treatment | 59.21 | 51.66 | 85.02 | 83.26 |
| (T1+T2) | 392 | 342 | 193 | 189 |
|  |  |  |  |  |
|  |  |  |  |  |
| Video treatment | 57.85 | 51.69 | 86.61 | 80.36 |
| (T1) | 188 | 168 | 97 | 90 |
|  |  |  |  |  |
|  |  |  |  |  |
| Video + Spouse treatment | 60.83 | 51.63 | 83.48 | 86.09 |
| (T2) | 205 | 174 | 96 | 99 |
|  |  |  |  |  |
|  |  |  |  |  |
| Control (T0) | 6.47 | 3.53 | 17.39 | 9.57 |
|  | 22 | 12 | 20 | 11 |
|  |  |  |  |  |
| Total | 1,002 | 1,002 | 342 | 342 |

# Appendix D: Uptake, by technology and crop

**Table D1. Estimates of treatment effects on sustained uptake, by technology and crop**

|  | Sustained uptake: by technology | | | | | | | |
| --- | --- | --- | --- | --- | --- | --- | --- | --- |
|  | **Any technology** | | **Row planting** | | **Precise seeding rate** | | **Urea top/side dressing** | |
| Pooled treatment | 0.0720*** |  | 0.0558*** |  | 0.0781*** |  | 0.0403** |  |
| (T1+T2) | (0.0227) |  | (0.0214) |  | (0.0199) |  | (0.0198) |  |
|  |  |  |  |  |  |  |  |  |
| Video treatment |  | 0.0869*** |  | 0.0648*** |  | 0.0923*** |  | 0.0418* |
| (T1) |  | (0.0273) |  | (0.0245) |  | (0.0247) |  | (0.0218) |
|  |  |  |  |  |  |  |  |  |
| Video + Spouse treatment |  | 0.0579** |  | 0.0472* |  | 0.0646*** |  | 0.0281 |
| (T2) |  | (0.0251) |  | (0.0244) |  | (0.0224) |  | (0.0223) |
|  |  |  |  |  |  |  |  |  |
| F Test |  | 1.226 |  | 0.555 |  | 1.219 |  | 0.363 |
| Prob F |  | 0.269 |  | 0.457 |  | 0.270 |  | 0.547 |
| Constant | 0.516*** | 0.516*** | 0.307*** | 0.307*** | 0.238*** | 0.239*** | 0.335*** | 0.307*** |
|  | (0.0293) | (0.0293) | (0.0247) | (0.0247) | (0.0263) | (0.0262) | (0.0257) | (0.0238) |
| Control mean | 0.489 | 0.489 | 0.318 | 0.318 | 0.214 | 0.214 | 0.345 | 0.345 |
| Observations | 2,119 | 2,119 | 2,119 | 2,119 | 2,119 | 2,119 | 2,119 | 2,345 |
| R-squared | 0.292 | 0.292 | 0.438 | 0.438 | 0.240 | 0.241 | 0.336 | 0.341 |
|  | **Sustained uptake: by crop** | | | | | | | |
|  | **Any crop** | | **Teff** | | **Wheat** | | **Maize** | |
| Pooled treatment | 0.0720*** |  | 0.0599** |  | 0.0596** |  | 0.0292 |  |
| (T1+T2) | (0.0227) |  | (0.0257) |  | (0.0237) |  | (0.0237) |  |
|  |  |  |  |  |  |  |  |  |
| Video treatment |  | 0.0869*** |  | 0.0602** |  | 0.0711** |  | 0.0516* |
| (T1) |  | (0.0273) |  | (0.0290) |  | (0.0276) |  | (0.0267) |
|  |  |  |  |  |  |  |  |  |
| Video + Spouse treatment |  | 0.0579** |  | 0.0595* |  | 0.0488* |  | 0.00754 |
| (T2) |  | (0.0251) |  | (0.0319) |  | (0.0275) |  | (0.0288) |
|  |  |  |  |  |  |  |  |  |
| F Test |  | 1.226 |  | 0.000391 |  | 0.619 |  | 2.283 |
| Prob F |  | 0.269 |  | 0.984 |  | 0.432 |  | 0.132 |
| Constant | 0.516*** | 0.516*** | 0.269*** | 0.269*** | 0.263*** | 0.264*** | 0.448*** | 0.449*** |
|  | (0.0293) | (0.0293) | (0.0325) | (0.0324) | (0.0303) | (0.0303) | (0.0314) | (0.0314) |
| Control mean | 0.489 | 0.489 | 0.237 | 0.237 | 0.248 | 0.248 | 0.452 | 0.452 |
| Observations | 2,119 | 2,119 | 1,498 | 1,498 | 1,442 | 1,442 | 1,301 | 1,301 |
| R-squared | 0.292 | 0.292 | 0.190 | 0.190 | 0.245 | 0.245 | 0.399 | 0.400 |

*Note*: Robust standard errors in parentheses, clustered at the kebele level. *Woreda* fixed effects. Controls for distance to nearest FTC (categories), whether household head received formal education, distance to nearest dry season road, distance to nearest all-weather road, distance to nearest marketplace, and distance to DA office/house. *** p<0.01, ** p<0.05, * p<0.1.

**Table D2. Estimates of treatment effects on cumulative uptake, by technology and crop**

|  | Cumulative uptake: by technology | | | | | | | |
| --- | --- | --- | --- | --- | --- | --- | --- | --- |
|  | **Any technology** | | **Row planting** | | **Precise seeding rate** | | **Urea top/side dressing** | |
| Pooled treatment | 0.0617*** |  | 0.0751*** |  | 0.0797*** |  | 0.0739*** |  |
| (T1+T2) | (0.0184) |  | (0.0234) |  | (0.0238) |  | (0.0226) |  |
|  |  |  |  |  |  |  |  |  |
| Video treatment |  | 0.0623*** |  | 0.0853*** |  | 0.0841*** |  | 0.0728*** |
| (T1) |  | (0.0214) |  | (0.0271) |  | (0.0267) |  | (0.0263) |
|  |  |  |  |  |  |  |  |  |
| Video + Spouse treatment |  | 0.0612*** |  | 0.0655** |  | 0.0756*** |  | 0.0749*** |
| (T2) |  | (0.0198) |  | (0.0267) |  | (0.0273) |  | (0.0251) |
|  |  |  |  |  |  |  |  |  |
| F Test |  | 0.00409 |  | 0.557 |  | 0.111 |  | 0.00689 |
| Prob F |  | 0.949 |  | 0.456 |  | 0.739 |  | 0.934 |
| Constant | 0.830*** | 0.830*** | 0.595*** | 0.596*** | 0.671*** | 0.671*** | 0.657*** | 0.656*** |
|  | (0.0234) | (0.0235) | (0.0284) | (0.0284) | (0.0314) | (0.0314) | (0.0297) | (0.0297) |
| Control mean | 0.826 | 0.826 | 0.566 | 0.566 | 0.639 | 0.639 | 0.669 | 0.669 |
| Observations | 2,119 | 2,119 | 2,119 | 2,119 | 2,119 | 2,119 | 2,119 | 2,119 |
| R-squared | 0.160 | 0.160 | 0.389 | 0.390 | 0.144 | 0.144 | 0.251 | 0.251 |
|  | **Cumulative uptake: by crop** | | | | | | | |
|  | **Any crop** | | **Teff** | | **Wheat** | | **Maize** | |
| Pooled treatment | 0.0617*** |  | 0.0737*** |  | 0.0796*** |  | 0.0443** |  |
| (T1+T2) | (0.0184) |  | (0.0246) |  | (0.0229) |  | (0.0188) |  |
|  |  |  |  |  |  |  |  |  |
| Video treatment |  | 0.0623*** |  | 0.0782*** |  | 0.0852*** |  | 0.0547*** |
| (T1) |  | (0.0214) |  | (0.0286) |  | (0.0267) |  | (0.0210) |
|  |  |  |  |  |  |  |  |  |
| Video + Spouse treatment |  | 0.0612*** |  | 0.0692** |  | 0.0744*** |  | 0.0343 |
| (T2) |  | (0.0198) |  | (0.0282) |  | (0.0261) |  | (0.0218) |
|  |  |  |  |  |  |  |  |  |
| F Test |  | 0.00409 |  | 0.101 |  | 0.170 |  | 0.993 |
| Prob F |  | 0.949 |  | 0.751 |  | 0.680 |  | 0.320 |
| Constant | 0.830*** | 0.830*** | 0.698*** | 0.698*** | 0.672*** | 0.672*** | 0.812*** | 0.812*** |
|  | (0.0234) | (0.0235) | (0.0323) | (0.0323) | (0.0327) | (0.0327) | (0.0272) | (0.0273) |
| Control mean | 0.826 | 0.826 | 0.676 | 0.676 | 0.643 | 0.643 | 0.831 | 0.831 |
| Observations | 2,119 | 2,119 | 1,498 | 1,498 | 1,442 | 1,442 | 1,301 | 1,301 |
| R-squared | 0.160 | 0.160 | 0.225 | 0.225 | 0.254 | 0.254 | 0.251 | 0.252 |

*Note*: Robust standard errors in parentheses, clustered at the kebele level. *Woreda* fixed effects. Controls for distance to nearest FTC (categories), whether household head received formal education, distance to nearest dry season road, distance to nearest all-weather road, distance to nearest marketplace, and distance to DA office/house. *** p<0.01, ** p<0.05, * p<0.1.

#

# Appendix E: Expansion on main results by crop

# Table E1. Estimates of treatment effects on technology adoption, by crop and year

|  | Uptake of any technology | | | | | | | |
| --- | --- | --- | --- | --- | --- | --- | --- | --- |
|  | **Any crop** | | **Teff** | | **Wheat** | | **Maize** | |
| *Panel A: meher 2017/18 (year 1)* | | | | | | |  |  |
| Pooled treatment | 0.0624*** |  | 0.0669** |  | 0.0796*** |  | 0.0173 |  |
| (T1+T2) | (0.0187) |  | (0.0260) |  | (0.0252) |  | (0.0187) |  |
| Video treatment |  | 0.0787*** |  | 0.0799*** |  | 0.102*** |  | 0.0314 |
| (T1) |  | (0.0226) |  | (0.0283) |  | (0.0289) |  | (0.0230) |
| Video + Spouse treatment |  | 0.0470** |  | 0.0538* |  | 0.0586** |  | 0.00381 |
| (T2) |  | (0.0208) |  | (0.0317) |  | (0.0289) |  | (0.0210) |
| F Test |  | 2.056 |  | 0.755 |  | 2.356 |  | 1.379 |
| Prob F |  | 0.153 |  | 0.386 |  | 0.126 |  | 0.241 |
| Constant | 0.651*** | 0.651*** | 0.497*** | 0.497*** | 0.489*** | 0.490*** | 0.725*** | 0.726*** |
|  | (0.0256) | (0.0256) | (0.0344) | (0.0344) | (0.0342) | (0.0341) | (0.0278) | (0.0279) |
| Control mean | 0.636 | 0.636 | 0.498 | 0.498 | 0.497 | 0.497 | 0.721 | 0.721 |
| Observations | 2,345 | 2,345 | 1,498 | 1,498 | 1,442 | 1,442 | 1,301 | 1,301 |
| R-squared | 0.285 | 0.286 | 0.282 | 0.283 | 0.310 | 0.311 | 0.389 | 0.390 |
| *Panel B: meher 2018/19 (year 2)* | | | | | | |  |  |
| Pooled treatment | 0.0739*** |  | 0.0589* |  | 0.0585** |  | 0.108*** |  |
| (T1+T2) | (0.0249) |  | (0.0309) |  | (0.0289) |  | (0.0251) |  |
| Video treatment |  | 0.0816*** |  | 0.0482 |  | 0.0581* |  | 0.122*** |
| (T1) |  | (0.0287) |  | (0.0364) |  | (0.0337) |  | (0.0275) |
| Video + Spouse treatment |  | 0.0666** |  | 0.0699** |  | 0.0590* |  | 0.0948*** |
| (T2) |  | (0.0274) |  | (0.0343) |  | (0.0326) |  | (0.0286) |
| F Test |  | 0.346 |  | 0.403 |  | 0.000753 |  | 1.181 |
| Prob F |  | 0.557 |  | 0.526 |  | 0.978 |  | 0.278 |
| Constant | 0.686*** | 0.686*** | 0.596*** | 0.595*** | 0.568*** | 0.568*** | 0.747*** | 0.749*** |
|  | (0.0299) | (0.0299) | (0.0405) | (0.0404) | (0.0366) | (0.0366) | (0.0353) | (0.0353) |
| Control mean | 0.664 | 0.664 | 0.555 | 0.555 | 0.515 | 0.515 | 0.767 | 0.767 |
| Observations | 2,119 | 2,119 | 1,268 | 1,268 | 1,315 | 1,315 | 1,053 | 1,053 |
| R-squared | 0.170 | 0.170 | 0.193 | 0.193 | 0.177 | 0.177 | 0.219 | 0.220 |

*Note*: Robust standard errors in parentheses, clustered at the kebele level. *Woreda* fixed effects. Controls for distance to nearest FTC (categories), whether household head received formal education, distance to nearest dry season road, distance to nearest all-weather road, distance to nearest marketplace, and distance to DA office/house. *** p<0.01, ** p<0.05, * p<0.1.

**Table E2. Estimates for treatment effects on plot-level yield, by crop**

|  | **Self-reported yield, plot level** | | | | | |
| --- | --- | --- | --- | --- | --- | --- |
|  | **Teff** | | **Wheat** | | **Maize** | |
| *Panel A: meher 2017/18 (year 1)* | | | | | | |
|  |  |  |  |  |  |  |
| Pooled treatment | 0.469 |  | -0.168 |  | 0.539 |  |
| (T1+T2) | (0.428) |  | (0.721) |  | (1.189) |  |
|  |  |  |  |  |  |  |
| Video treatment |  | 0.609 |  | -0.126 |  | 1.224 |
| (T1) |  | (0.491) |  | (0.881) |  | (1.286) |
|  |  |  |  |  |  |  |
| Video + Spouse treatment |  | 0.337 |  | -0.209 |  | -0.128 |
| (T2) |  | (0.485) |  | (0.832) |  | (1.338) |
|  |  |  |  |  |  |  |
| F Test |  | 0.333 |  | 0.00807 |  | 1.448 |
| Prob F |  | 0.564 |  | 0.928 |  | 0.230 |
| Constant | 9.031*** | 9.027*** | 18.83*** | 18.83*** | 26.23*** | 26.26*** |
|  | (0.486) | (0.485) | (0.845) | (0.845) | (1.321) | (1.326) |
| Control mean | 8.920 | 8.920 | 19.86 | 19.86 | 26.10 | 26.10 |
| Observations | 2,516 | 2,516 | 2,030 | 2,030 | 1,645 | 1,645 |
| R-squared | 0.169 | 0.169 | 0.259 | 0.259 | 0.277 | 0.277 |
| *Panel B: meher 2018/19 (year 2)* | | | | | | |
|  |  |  |  |  |  |  |
| Pooled treatment | 0.455 |  | -0.0742 |  | 0.492 |  |
| (T1+T2) | (0.503) |  | (0.801) |  | (1.166) |  |
|  |  |  |  |  |  |  |
| Video treatment |  | 0.625 |  | 0.0369 |  | 1.602 |
| (T1) |  | (0.551) |  | (0.990) |  | (1.244) |
|  |  |  |  |  |  |  |
| Video + Spouse treatment |  | 0.276 |  | -0.189 |  | -0.588 |
| (T2) |  | (0.591) |  | (0.841) |  | (1.352) |
|  |  |  |  |  |  |  |
| F Test |  | 0.416 |  | 0.0625 |  | 3.538 |
| Prob F |  | 0.519 |  | 0.803 |  | 0.0611 |
| Constant | 8.430*** | 8.443*** | 19.03*** | 19.03*** | 24.83*** | 24.95*** |
|  | (0.554) | (0.554) | (1.048) | (1.047) | (1.549) | (1.555) |
| Control mean | 8.528 | 8.528 | 19.71 | 19.71 | 25.33 | 25.33 |
| Observations | 2,240 | 2,240 | 1,882 | 1,882 | 1,346 | 1,346 |
| R-squared | 0.249 | 0.250 | 0.361 | 0.361 | 0.315 | 0.317 |

*Note*: Robust standard errors in parentheses, clustered at the kebele level. *Woreda* fixed effects. Upper end of the yield distribution winsorized at the 1 percent level. Controls for distance to nearest FTC (categories), whether household head received formal education, distance to nearest dry season road, distance to nearest all-weather road, distance to nearest marketplace, and distance to DA office/house. *** p<0.01, ** p<0.05, * p<0.1.

**Table E3. Estimates for treatment effects on plot-level yield based on GPS measured area, by crop**

|  | **Yield based on GPS measured area, plot level** | | | | | |
| --- | --- | --- | --- | --- | --- | --- |
|  | **Teff** | | **Wheat** | | **Maize** | |
| *Panel A: meher 2017/18 (year 1)* | | | | | | |
|  |  |  |  |  |  |  |
| Pooled treatment | 1.484 |  | -0.522 |  | 3.284 |  |
| (T1+T2) | (1.140) |  | (2.139) |  | (2.567) |  |
|  |  |  |  |  |  |  |
| Video treatment |  | 2.352* |  | -0.422 |  | 3.544 |
| (T1) |  | (1.309) |  | (2.564) |  | (2.832) |
|  |  |  |  |  |  |  |
| Video + Spouse treatment |  | 0.653 |  | -0.613 |  | 3.031 |
| (T2) |  | (1.219) |  | (2.226) |  | (3.234) |
|  |  |  |  |  |  |  |
| F Test |  | 2.370 |  | 0.00799 |  | 0.0247 |
| Prob F |  | 0.125 |  | 0.929 |  | 0.875 |
| Constant | 9.986*** | 10.02*** | 22.85*** | 22.86*** | 32.75*** | 32.78*** |
|  | (1.221) | (1.221) | (2.641) | (2.640) | (3.265) | (3.294) |
| Control mean | 10.63 | 10.63 | 26.29 | 26.29 | 35.44 | 35.44 |
| Observations | 787 | 787 | 799 | 799 | 829 | 829 |
| R-squared | 0.104 | 0.106 | 0.117 | 0.117 | 0.116 | 0.116 |
| *Panel B: meher 2018/19 (year 2)* | | | | | | |
|  |  |  |  |  |  |  |
| Pooled treatment | 1.389 |  | 1.436 |  | 0.0563 |  |
| (T1+T2) | (1.851) |  | (2.768) |  | (4.179) |  |
|  |  |  |  |  |  |  |
| Video treatment |  | 0.783 |  | 2.940 |  | -0.534 |
| (T1) |  | (2.067) |  | (3.656) |  | (5.038) |
|  |  |  |  |  |  |  |
| Video + Spouse treatment |  | 2.046 |  | -0.162 |  | 0.590 |
| (T2) |  | (2.162) |  | (2.844) |  | (5.008) |
|  |  |  |  |  |  |  |
| F Test |  | 0.383 |  | 0.756 |  | 0.0407 |
| Prob F |  | 0.536 |  | 0.385 |  | 0.840 |
| Constant | 17.41*** | 17.32*** | 28.22*** | 28.17*** | 41.02*** | 40.97*** |
|  | (2.436) | (2.440) | (3.557) | (3.571) | (6.071) | (6.123) |
| Control mean | 16.43 | 16.43 | 27.91 | 27.91 | 35.39 | 35.39 |
| Observations | 679 | 679 | 681 | 681 | 795 | 795 |
| R-squared | 0.105 | 0.105 | 0.120 | 0.122 | 0.083 | 0.083 |

*Note*: Robust standard errors in parentheses, clustered at the kebele level. *Woreda* fixed effects. Upper end of the yield distribution winsorized at the 1 percent level. Controls for distance to nearest FTC (categories), whether household head received formal education, distance to nearest dry season road, distance to nearest all-weather road, distance to nearest marketplace, and distance to DA office/house. *** p<0.01, ** p<0.05, * p<0.1.

# Appendix F: Replicating the main results using a restricted sample (development groups with access to video-mediated extension)

**Table F1. Sample size, by survey round (restricted sample)**

| Sample | Video treatment  (T1) | Video + Spouse treatment  (T2) | Control (T0) | Total |
| --- | --- | --- | --- | --- |
| Total number of *woreda*s | 30 | (T2) | 30 | 30 |
| Total number of *kebele*s: |  |  |  |  |
| *year 1* | 105 | 111 | 115 | 331 |
| *year 2* | 99 | 106 | 115 | 320 |
| Total number of households: |  |  |  |  |
| *year 1* | 593 | 633 | 792 | 2,018 |
| *year 2* | 479 | 521 | 792 | 1,792 |

*Note*: Random assignment of *kebeles* to treatment and control groups was stratified by *woreda*. This implies that each of the 30 *woredas* selected for the study contained *kebeles* assigned to both treatment groups and the control group. For this reason, a total of 30 *woredas* are shown in the last column.

**Table F2. Uptake trends by treatment group (percentage of total in each respective treatment group) (restricted sample)**

|  | | | **Row planting** | | **Precise seeding rate** | | **Urea top/side dressing** | | **Any technology** | |
| --- | --- | --- | --- | --- | --- | --- | --- | --- | --- | --- |
|  |  |  | Year 1 (%) | | | | | | | |
|  |  |  | No | Yes | No | Yes | No | Yes | No | Yes |
| Video treatment (T1) | Year 2 (%) | No | 35 | 16 | 27 | 24 | 26 | 22 | 9 | 20 |
|  |  | Yes | 12 | 36 | 19 | 30 | 14 | 38 | 15 | 56 |
| Video + Spouse treatment (T2) |  | No | 39 | 15 | 30 | 24 | 26 | 23 | 13 | 20 |
|  |  | Yes | 12 | 34 | 19 | 27 | 15 | 37 | 15 | 52 |
| Pooled treatment (T1+T2) |  | No | 37 | 16 | 28 | 24 | 26 | 23 | 11 | 20 |
|  |  | Yes | 12 | 35 | 19 | 28 | 14 | 37 | 15 | 54 |
| Control (T0) |  | No | 46 | 14 | 39 | 21 | 37 | 17 | 21 | 20 |
|  |  | Yes | 11 | 29 | 20 | 19 | 15 | 31 | 16 | 44 |

*Note*: Treatment group sample restricted to households in a development group that received a DA visit in either year 1 or year 2.

**Table F3. Estimates of treatment effects on technology uptake, by technology and year (restricted sample)**

|  | Uptake of technology | | | | | | | |
| --- | --- | --- | --- | --- | --- | --- | --- | --- |
|  | **Any technology** | | **Row planting** | | **Precise seeding rate** | | **Urea top/side dressing** | |
| *Panel A: meher 2017/18 (year 1)* | | | | | | |  |  |
|  |  |  |  |  |  |  |  |  |
| Pooled treatment | 0.0887*** |  | 0.0734*** |  | 0.117*** |  | 0.106*** |  |
| (T1+T2) | (0.0200) |  | (0.0219) |  | (0.0236) |  | (0.0214) |  |
|  |  |  |  |  |  |  |  |  |
| Video treatment |  | 0.108*** |  | 0.0929*** |  | 0.129*** |  | 0.105*** |
| (T1) |  | (0.0246) |  | (0.0273) |  | (0.0291) |  | (0.0251) |
|  |  |  |  |  |  |  |  |  |
| Video + Spouse treatment |  | 0.0710*** |  | 0.0553** |  | 0.106*** |  | 0.106*** |
| (T2) |  | (0.0226) |  | (0.0241) |  | (0.0273) |  | (0.0255) |
|  |  |  |  |  |  |  |  |  |
| F Test |  | 2.167 |  | 1.922 |  | 0.574 |  | 0.00176 |
| Prob F |  | 0.142 |  | 0.167 |  | 0.449 |  | 0.967 |
| Constant | 0.670*** | 0.671*** | 0.486*** | 0.487*** | 0.458*** | 0.458*** | 0.477*** | 0.477*** |
|  | (0.0271) | (0.0271) | (0.0279) | (0.0279) | (0.0325) | (0.0325) | (0.0274) | (0.0274) |
| Control mean | 0.636 | 0.636 | 0.428 | 0.428 | 0.405 | 0.405 | 0.485 | 0.485 |
| Observations | 2,018 | 2,018 | 2,018 | 2,018 | 2,018 | 2,018 | 2,018 | 2,018 |
| R-squared | 0.263 | 0.264 | 0.429 | 0.430 | 0.159 | 0.159 | 0.310 | 0.310 |
| *Panel B: meher 2018/19 (year 2)* | | | | | | |  |  |
|  |  |  |  |  |  |  |  |  |
| Pooled treatment | 0.143*** |  | 0.116*** |  | 0.117*** |  | 0.101*** |  |
| (T1+T2) | (0.0244) |  | (0.0250) |  | (0.0262) |  | (0.0262) |  |
|  |  |  |  |  |  |  |  |  |
| Video treatment |  | 0.157*** |  | 0.121*** |  | 0.130*** |  | 0.105*** |
| (T1) |  | (0.0288) |  | (0.0301) |  | (0.0312) |  | (0.0311) |
|  |  |  |  |  |  |  |  |  |
| Video + Spouse treatment |  | 0.130*** |  | 0.111*** |  | 0.105*** |  | 0.0973*** |
| (T2) |  | (0.0266) |  | (0.0287) |  | (0.0294) |  | (0.0300) |
|  |  |  |  |  |  |  |  |  |
| F Test |  | 1.077 |  | 0.106 |  | 0.678 |  | 0.0563 |
| Prob F |  | 0.300 |  | 0.745 |  | 0.411 |  | 0.813 |
| Constant | 0.693*** | 0.693*** | 0.448*** | 0.449*** | 0.446*** | 0.447*** | 0.514*** | 0.514*** |
|  | (0.0294) | (0.0294) | (0.0299) | (0.0300) | (0.0352) | (0.0352) | (0.0327) | (0.0327) |
| Control mean | 0.664 | 0.664 | 0.440 | 0.440 | 0.441 | 0.441 | 0.507 | 0.507 |
| Observations | 1,656 | 1,656 | 1,656 | 1,656 | 1,656 | 1,656 | 1,656 | 1,656 |
| R-squared | 0.180 | 0.181 | 0.378 | 0.378 | 0.224 | 0.224 | 0.238 | 0.238 |

*Note*: Robust standard errors in parentheses, clustered at the kebele level. Woreda fixed effects. Controls for distance to nearest FTC (categories), whether household head received formal education, distance to nearest dry season road, distance to nearest all-weather road, distance to nearest marketplace, and distance to DA office/house. *** p<0.01, ** p<0.05, * p<0.1.

**Table F4. Estimates of treatment effects on sustained uptake, by technology (restricted sample)**

|  | Sustained adoption | | | | | | | |
| --- | --- | --- | --- | --- | --- | --- | --- | --- |
|  | **Any technology** | | **Row planting** | | **Precise seeding rate** | | **Urea top/side dressing** | |
|  |  |  |  |  |  |  |  |  |
| Pooled treatment | 0.0902*** |  | 0.0601*** |  | 0.0958*** |  | 0.0563*** |  |
| (T1+T2) | (0.0235) |  | (0.0223) |  | (0.0209) |  | (0.0207) |  |
|  |  |  |  |  |  |  |  |  |
| Video treatment |  | 0.113*** |  | 0.0746*** |  | 0.116*** |  | 0.0668*** |
| (T1) |  | (0.0288) |  | (0.0263) |  | (0.0263) |  | (0.0235) |
|  |  |  |  |  |  |  |  |  |
| Video + Spouse treatment |  | 0.0686*** |  | 0.0465* |  | 0.0767*** |  | 0.0464* |
| (T2) |  | (0.0261) |  | (0.0251) |  | (0.0238) |  | (0.0252) |
|  |  |  |  |  |  |  |  |  |
| F Test |  | 2.496 |  | 1.208 |  | 2.037 |  | 0.626 |
| Prob F |  | 0.115 |  | 0.272 |  | 0.154 |  | 0.430 |
| Constant | 0.525*** | 0.527*** | 0.322*** | 0.323*** | 0.240*** | 0.241*** | 0.339*** | 0.340*** |
|  | (0.0310) | (0.0310) | (0.0259) | (0.0258) | (0.0276) | (0.0275) | (0.0271) | (0.0271) |
| Control mean | 0.489 | 0.489 | 0.318 | 0.318 | 0.214 | 0.214 | 0.345 | 0.345 |
| Observations | 1,921 | 1,921 | 1,921 | 1,921 | 1,921 | 1,921 | 1,921 | 1,921 |
| R-squared | 0.280 | 0.281 | 0.443 | 0.444 | 0.247 | 0.249 | 0.338 | 0.338 |

*Note*: Robust standard errors in parentheses, clustered at the kebele level. Woreda fixed effects. Controls for distance to nearest FTC (categories), whether household head received formal education, distance to nearest dry season road, distance to nearest all-weather road, distance to nearest marketplace, and distance to DA office/house. *** p<0.01, ** p<0.05, * p<0.1.

**Table F5. Estimates of treatment effects on cumulative uptake, by technology (restricted sample)**

|  | Cumulative adoption | | | | | | | |
| --- | --- | --- | --- | --- | --- | --- | --- | --- |
|  | **Any technology** | | **Row planting** | | **Precise seeding rate** | | **Urea top/side dressing** | |
| Pooled treatment | 0.0843*** |  | 0.0859*** |  | 0.101*** |  | 0.0985*** |  |
| (T1+T2) | (0.0177) |  | (0.0238) |  | (0.0237) |  | (0.0222) |  |
|  |  |  |  |  |  |  |  |  |
| Video treatment |  | 0.0969*** |  | 0.104*** |  | 0.111*** |  | 0.104*** |
| (T1) |  | (0.0200) |  | (0.0276) |  | (0.0269) |  | (0.0258) |
|  |  |  |  |  |  |  |  |  |
| Video + Spouse treatment |  | 0.0724*** |  | 0.0685** |  | 0.0913*** |  | 0.0931*** |
| (T2) |  | (0.0199) |  | (0.0273) |  | (0.0277) |  | (0.0250) |
|  |  |  |  |  |  |  |  |  |
| F Test |  | 1.768 |  | 1.710 |  | 0.549 |  | 0.202 |
| Prob F |  | 0.184 |  | 0.192 |  | 0.459 |  | 0.654 |
| Constant | 0.835*** | 0.836*** | 0.617*** | 0.618*** | 0.676*** | 0.677*** | 0.667*** | 0.667*** |
|  | (0.0230) | (0.0231) | (0.0299) | (0.0299) | (0.0319) | (0.0319) | (0.0295) | (0.0296) |
| Control mean | 0.826 | 0.826 | 0.566 | 0.566 | 0.639 | 0.639 | 0.669 | 0.669 |
| Observations | 1,921 | 1,921 | 1,921 | 1,921 | 1,921 | 1,921 | 1,921 | 1,921 |
| R-squared | 0.140 | 0.141 | 0.396 | 0.397 | 0.136 | 0.136 | 0.255 | 0.255 |

*Source*: Authors’ calculations.

*Note*: Robust standard errors in parentheses, clustered at the kebele level. Woreda fixed effects. Controls for distance to nearest FTC (categories), whether household head received formal education, distance to nearest dry season road, distance to nearest all-weather road, distance to nearest marketplace, and distance to DA office/house. *** p<0.01, ** p<0.05, * p<0.1.

**Table F6. Estimates for treatment effects on household-level yield, by crop (restricted sample)**

|  | **Self-reported yield, household level** | | | | | |
| --- | --- | --- | --- | --- | --- | --- |
|  | **Teff** | | **Wheat** | | **Maize** | |
| *Panel A: meher 2017/18 (year 1)* | | | | | | |
|  | | | | | | |
| Pooled treatment | 0.937** |  | 0.687 |  | 1.389 |  |
| (T1+T2) | (0.411) |  | (0.802) |  | (1.176) |  |
|  |  |  |  |  |  |  |
| Video treatment |  | 1.353** |  | 0.286 |  | 2.224* |
| (T1) |  | (0.543) |  | (1.035) |  | (1.272) |
|  |  |  |  |  |  |  |
| Video + Spouse treatment |  | 0.520 |  | 1.056 |  | 0.598 |
| (T2) |  | (0.427) |  | (0.931) |  | (1.353) |
|  |  |  |  |  |  |  |
| F Test |  | 2.409 |  | 0.461 |  | 1.877 |
| Prob F |  | 0.122 |  | 0.498 |  | 0.172 |
| Constant | 8.674*** | 8.679*** | 18.07*** | 18.05*** | 24.83*** | 24.85*** |
|  | (0.515) | (0.516) | (0.904) | (0.906) | (1.307) | (1.310) |
| Control mean | 8.447 | 8.447 | 18.74 | 18.74 | 23.82 | 23.82 |
| Observations | 1,278 | 1,278 | 1,243 | 1,243 | 1,128 | 1,128 |
| R-squared | 0.202 | 0.205 | 0.255 | 0.255 | 0.303 | 0.304 |
| *Panel B: meher 2018/19 (year 2)* | | | | | | |
|  | | | | | | |
| Pooled treatment | 0.397 |  | 0.274 |  | 1.238 |  |
| (T1+T2) | (0.442) |  | (0.771) |  | (1.181) |  |
|  |  |  |  |  |  |  |
| Video treatment |  | 0.453 |  | 0.407 |  | 1.881 |
| (T1) |  | (0.512) |  | (1.026) |  | (1.304) |
|  |  | 0.343 |  | 0.146 |  | 0.675 |
| Video + Spouse treatment |  | (0.527) |  | (0.811) |  | (1.437) |
| (T2) |  | 0.0401 |  | 0.0660 |  | 0.733 |
|  |  |  |  |  |  |  |
| Prob F |  | 0.841 |  | 0.797 |  | 0.393 |
| Constant | 8.087*** | 8.090*** | 17.45*** | 17.46*** | 23.65*** | 23.68*** |
|  | (0.482) | (0.482) | (0.974) | (0.973) | (1.485) | (1.486) |
| Control mean | 8.280 | 8.280 | 18.09 | 18.09 | 23.80 | 23.80 |
| Observations | 954 | 954 | 1,039 | 1,039 | 818 | 818 |
| R-squared | 0.300 | 0.300 | 0.417 | 0.417 | 0.358 | 0.359 |

*Source*: Authors’ calculations.

*Note*: Robust standard errors in parentheses, clustered at the kebele level. Woreda fixed effects. Upper end of the yield distribution winsorized at the 1 percent level. Controls for distance to nearest FTC (categories), whether household head received formal education, distance to nearest dry season road, distance to nearest all-weather road, distance to nearest marketplace, and distance to DA office/house. *** p<0.01, ** p<0.05, * p<0.1.

**Table F7. Estimates of treatment effects on extension access for the household head, any technology (restricted sample)**

|  | Extension access for household head, any technology | | | | | | | |
| --- | --- | --- | --- | --- | --- | --- | --- | --- |
|  | **DA provided**  **advice/training** | | **No. of times DA**  **provided advice/training** | | **DA visited plot** | | **No. of plots**  **visited by DA** | |
| *Panel A: meher 2017/18 (year 1)* | | | | | | |  |  |
| Pooled treatment | 0.283*** |  | 1.405*** |  | 0.0992*** |  | 0.227*** |  |
| (T1+T2) | (0.0208) |  | (0.183) |  | (0.0237) |  | (0.0551) |  |
| Video treatment |  | 0.290*** |  | 1.580*** |  | 0.100*** |  | 0.234*** |
| (T1) |  | (0.0241) |  | (0.243) |  | (0.0290) |  | (0.0682) |
| Video + Spouse treatment |  | 0.275*** |  | 1.244*** |  | 0.0983*** |  | 0.220*** |
| (T2) |  | (0.0230) |  | (0.203) |  | (0.0263) |  | (0.0647) |
| F Test |  | 0.458 |  | 1.726 |  | 0.00431 |  | 0.0367 |
| Prob F |  | 0.499 |  | 0.190 |  | 0.948 |  | 0.848 |
| Constant | 0.542*** | 0.542*** | 2.573*** | 2.578*** | 0.400*** | 0.400*** | 0.822*** | 0.822*** |
|  | (0.0282) | (0.0282) | (0.226) | (0.226) | (0.0313) | (0.0313) | (0.0754) | (0.0754) |
| Control mean | 0.472 | 0.472 | 2.306 | 2.306 | 0.340 | 0.340 | 0.676 | 0.676 |
| Observations | 2,018 | 2,018 | 2,018 | 2,018 | 2,018 | 2,018 | 2,017 | 2,017 |
| R-squared | 0.311 | 0.311 | 0.264 | 0.265 | 0.244 | 0.244 | 0.306 | 0.306 |
| *Panel B: meher 2018/19 (year 2)* | | | | | | |  |  |
| Pooled treatment | 0.282*** |  | 1.120*** |  | 0.0945*** |  | 0.137*** |  |
| (T1+T2) | (0.0252) |  | (0.126) |  | (0.0240) |  | (0.0469) |  |
| Video treatment |  | 0.289*** |  | 1.186*** |  | 0.0959*** |  | 0.125** |
| (T1) |  | (0.0284) |  | (0.152) |  | (0.0294) |  | (0.0608) |
| Video + Spouse treatment |  | 0.276*** |  | 1.059*** |  | 0.0932*** |  | 0.149*** |
| (T2) |  | (0.0277) |  | (0.145) |  | (0.0284) |  | (0.0556) |
| F Test |  | 0.303 |  | 0.630 |  | 0.00685 |  | 0.125 |
| Prob F |  | 0.583 |  | 0.428 |  | 0.934 |  | 0.724 |
| Constant | 0.492*** | 0.492*** | 1.536*** | 1.539*** | 0.314*** | 0.314*** | 0.612*** | 0.612*** |
|  | (0.0314) | (0.0315) | (0.195) | (0.195) | (0.0308) | (0.0308) | (0.0675) | (0.0675) |
| Control mean | 0.426 | 0.426 | 1.297 | 1.297 | 0.246 | 0.246 | 0.446 | 0.446 |
| Observations | 1,656 | 1,656 | 1,656 | 1,656 | 1,656 | 1,656 | 1,654 | 1,654 |
| R-squared | 0.230 | 0.230 | 0.185 | 0.185 | 0.209 | 0.209 | 0.239 | 0.239 |

*Note*: Robust standard errors in parentheses, clustered at the kebele level. Woreda fixed effects. Controls for distance to nearest FTC (categories), whether household head received formal education, distance to nearest dry season road, distance to nearest all-weather road, distance to nearest marketplace, and distance to DA office/house. *** p<0.01, ** p<0.05, * p<0.1.

**Table F8. Estimates of treatment effects on extension access for the spouse, any technology (restricted sample)**

|  | Extension access for spouse, any technology | | | |
| --- | --- | --- | --- | --- |
|  | **DA provided**  **advice/training** | | **No. of times DA**  **provided advice/training** | |
| *Panel A: meher 2017/18 (year 1)* | | | | |
|  | | | | |
| Pooled treatment | 0.384*** |  | 1.161*** |  |
| (T1+T2) | (0.0266) |  | (0.151) |  |
|  |  |  |  |  |
| Video treatment |  | 0.369*** |  | 1.229*** |
| (T1) |  | (0.0326) |  | (0.206) |
|  |  |  |  |  |
| Video + Spouse treatment |  | 0.399*** |  | 1.099*** |
| (T2) |  | (0.0303) |  | (0.170) |
|  |  |  |  |  |
| F Test |  | 0.806 |  | 0.333 |
| Prob F |  | 0.370 |  | 0.565 |
| Constant | 0.332*** | 0.331*** | 1.065*** | 1.070*** |
|  | (0.0336) | (0.0339) | (0.162) | (0.161) |
| Control mean | 0.245 | 0.245 | 0.801 | 0.801 |
| Observations | 1,109 | 1,109 | 1,109 | 1,109 |
| R-squared | 0.375 | 0.375 | 0.284 | 0.284 |
| *Panel B: meher 2018/19 (year 2)* | | | | |
|  |  |  |  |  |
| Pooled treatment | 0.392*** |  | 0.858*** |  |
| (T1+T2) | (0.0326) |  | (0.128) |  |
|  |  |  |  |  |
| Video treatment |  | 0.384*** |  | 0.794*** |
| (T1) |  | (0.0398) |  | (0.158) |
|  |  |  |  |  |
| Video + Spouse treatment |  | 0.400*** |  | 0.926*** |
| (T2) |  | (0.0411) |  | (0.174) |
|  |  |  |  |  |
| F Test |  | 0.107 |  | 0.390 |
| Prob F |  | 0.744 |  | 0.533 |
| Constant | 0.276*** | 0.276*** | 0.816*** | 0.816*** |
|  | (0.0374) | (0.0375) | (0.168) | (0.168) |
| Control mean | 0.194 | 0.194 | 0.545 | 0.545 |
| Observations | 913 | 913 | 913 | 913 |
| R-squared | 0.349 | 0.349 | 0.236 | 0.237 |

*Source*: Authors’ calculations.

*Note*: Robust standard errors in parentheses, clustered at the kebele level. Woreda fixed effects. Controls for distance to nearest FTC (categories), whether household head received formal education, distance to nearest dry season road, distance to nearest all-weather road, distance to nearest marketplace, and distance to DA office/house. *** p<0.01, ** p<0.05, * p<0.1.

**Table F9. Estimates for treatment effects on knowledge of subject technologies (restricted sample)**

|  | Knowledge score, percentage | | | |
| --- | --- | --- | --- | --- |
|  | **Head of the household** | | **Spouse** | |
| *Panel A: meher 2017/18 (year 1)* |  | |  | |
|  |  |  |  |  |
| Pooled treatment | 2.072*** |  | 1.690* |  |
| (T1+T2) | (0.549) |  | (0.867) |  |
|  |  |  |  |  |
| Video treatment |  | 1.825*** |  | 1.320 |
| (T1) |  | (0.661) |  | (0.983) |
|  |  |  |  |  |
| Video + Spouse treatment |  | 2.299*** |  | 2.021* |
| (T2) |  | (0.645) |  | (1.074) |
|  |  |  |  |  |
| F Test |  | 0.449 |  | 0.391 |
| Prob F |  | 0.503 |  | 0.532 |
| Constant | 40.96*** | 40.95*** | 38.18*** | 38.16*** |
|  | (0.719) | (0.720) | (1.020) | (1.020) |
| Control mean | 39.93 | 39.93 | 35.31 | 35.31 |
| Observations | 2,018 | 2,018 | 1,109 | 1,109 |
| R-squared | 0.180 | 0.180 | 0.233 | 0.234 |
| *Panel B: meher 2018/19 (year 2)* |  |  |  |  |
|  |  |  |  |  |
| Pooled treatment | 0.729 |  | 2.081** |  |
| (T1+T2) | (0.571) |  | (0.936) |  |
|  |  |  |  |  |
| Video treatment |  | 1.190* |  | 2.182** |
| (T1) |  | (0.678) |  | (1.072) |
|  |  |  |  |  |
| Video + Spouse treatment |  | 0.304 |  | 1.991* |
| (T2) |  | (0.676) |  | (1.156) |
|  |  |  |  |  |
| F Test |  | 1.475 |  | 0.0246 |
| Prob F |  | 0.225 |  | 0.875 |
| Constant | 45.90*** | 45.92*** | 37.69*** | 37.69*** |
|  | (0.790) | (0.792) | (1.111) | (1.113) |
| Control mean | 45.62 | 45.62 | 34.69 | 34.69 |
| Observations | 1,656 | 1,656 | 1,016 | 1,016 |
| R-squared | 0.141 | 0.142 | 0.235 | 0.235 |

*Source*: Authors’ calculations.

*Note*: Robust standard errors in parentheses, clustered at the kebele level. Woreda fixed effects. Controls for distance to nearest FTC (categories), whether household head received formal education, distance to nearest dry season road, distance to nearest all-weather road, distance to nearest marketplace, and distance to DA office/house. *** p<0.01, ** p<0.05, * p<0.1.

# Appendix G: Estimates of treatment effects on extension access, by crop and for spouses

**Table G1. Estimates of treatment effects on extension access, by crop**

|  | Extension access for household head  (DA provided advice/training), any technology | | | | | |
| --- | --- | --- | --- | --- | --- | --- |
|  | **Teff** | | **Wheat** | | **Maize** | |
| *Panel A: meher 2017/18 (year 1)* | | | | | | |
|  |  |  |  |  |  |  |
| Pooled treatment | 0.110*** |  | 0.160*** |  | 0.135*** |  |
| (T1+T2) | (0.0243) |  | (0.0253) |  | (0.0272) |  |
|  |  |  |  |  |  |  |
| Video treatment |  | 0.107*** |  | 0.156*** |  | 0.153*** |
| (T1) |  | (0.0282) |  | (0.0305) |  | (0.0304) |
|  |  |  |  |  |  |  |
| Video + Spouse treatment |  | 0.114*** |  | 0.164*** |  | 0.118*** |
| (T2) |  | (0.0279) |  | (0.0286) |  | (0.0316) |
|  |  |  |  |  |  |  |
| F Test |  | 0.0630 |  | 0.0807 |  | 1.321 |
| Prob F |  | 0.802 |  | 0.777 |  | 0.251 |
| Constant | 0.494*** | 0.494*** | 0.483*** | 0.483*** | 0.529*** | 0.530*** |
|  | (0.0351) | (0.0351) | (0.0348) | (0.0348) | (0.0346) | (0.0346) |
| Control mean | 0.455 | 0.455 | 0.425 | 0.425 | 0.493 | 0.493 |
| Observations | 1,498 | 1,498 | 1,442 | 1,442 | 1,301 | 1,301 |
| R-squared | 0.343 | 0.343 | 0.375 | 0.375 | 0.358 | 0.359 |
| *Panel B: meher 2018/19 (year 2)* | | | | | | |
|  |  |  |  |  |  |  |
| Pooled treatment | 0.0605* |  | 0.0938*** |  | 0.0690* |  |
| (T1+T2) | (0.0324) |  | (0.0308) |  | (0.0354) |  |
|  |  |  |  |  |  |  |
| Video treatment |  | 0.0502 |  | 0.0806** |  | 0.0665* |
| (T1) |  | (0.0387) |  | (0.0370) |  | (0.0393) |
|  |  |  |  |  |  |  |
| Video + Spouse treatment |  | 0.0710* |  | 0.107*** |  | 0.0714* |
| (T2) |  | (0.0365) |  | (0.0338) |  | (0.0419) |
|  |  |  |  |  |  |  |
| F Test |  | 0.295 |  | 0.557 |  | 0.0148 |
| Prob F |  | 0.587 |  | 0.456 |  | 0.903 |
| Constant | 0.429*** | 0.428*** | 0.436*** | 0.436*** | 0.486*** | 0.485*** |
|  | (0.0400) | (0.0400) | (0.0387) | (0.0388) | (0.0429) | (0.0429) |
| Control mean | 0.365 | 0.365 | 0.380 | 0.380 | 0.445 | 0.445 |
| Observations | 1,268 | 1,268 | 1,315 | 1,315 | 1,053 | 1,053 |
| R-squared | 0.205 | 0.206 | 0.206 | 0.207 | 0.229 | 0.229 |

*Note*: Robust standard errors in parentheses, clustered at the kebele level. Woreda fixed effects. Controls for distance to nearest FTC (categories), whether household head received formal education, distance to nearest dry season road, distance to nearest all-weather road, distance to nearest marketplace, and distance to DA office/house. *** p<0.01, ** p<0.05, * p<0.1.

**Table G2. Estimates of treatment effects on extension access for the spouse, any technology**

|  | Extension access for spouse, any technology | | | |
| --- | --- | --- | --- | --- |
|  | **DA provided**  **advice/training** | | **No. of times DA**  **provided advice/training** | |
| *Panel A: meher 2017/18 (year 1)* | | | | |
|  |  |  |  |  |
| Pooled treatment | 0.0614*** |  | 0.231** |  |
| (T1+T2) | (0.0201) |  | (0.0941) |  |
|  |  |  |  |  |
| Video treatment |  | 0.0442* |  | 0.241** |
| (T1) |  | (0.0237) |  | (0.117) |
|  |  |  |  |  |
| Video + Spouse treatment |  | 0.0778*** |  | 0.222** |
| (T2) |  | (0.0233) |  | (0.101) |
|  |  |  |  |  |
| F Test |  | 1.937 |  | 0.0272 |
| Prob F |  | 0.165 |  | 0.869 |
| Constant | 0.290*** | 0.289*** | 0.896*** | 0.896*** |
|  | (0.0293) | (0.0294) | (0.125) | (0.125) |
| Control mean | 0.245 | 0.245 | 0.801 | 0.801 |
| Observations | 1,839 | 1,839 | 1,839 | 1,839 |
| R-squared | 0.274 | 0.275 | 0.261 | 0.261 |
| *Panel B: meher 2018/19 (year 2)* | | | | |
|  |  |  |  |  |
| Pooled treatment | 0.0237 |  | 0.0319 |  |
| (T1+T2) | (0.0213) |  | (0.0688) |  |
|  |  |  |  |  |
| Video treatment |  | 0.0291 |  | 0.0285 |
| (T1) |  | (0.0251) |  | (0.0770) |
|  |  |  |  |  |
| Video + Spouse treatment |  | 0.0186 |  | 0.0352 |
| (T2) |  | (0.0241) |  | (0.0818) |
|  |  |  |  |  |
| F Test |  | 0.181 |  | 0.00708 |
| Prob F |  | 0.671 |  | 0.933 |
| Constant | 0.227*** | 0.227*** | 0.645*** | 0.645*** |
|  | (0.0283) | (0.0283) | (0.108) | (0.108) |
| Control mean | 0.194 | 0.194 | 0.545 | 0.545 |
| Observations | 1,686 | 1,686 | 1,686 | 1,686 |
| R-squared | 0.199 | 0.199 | 0.191 | 0.191 |

*Note*: Robust standard errors in parentheses, clustered at the kebele level. Woreda fixed effects. Controls for distance to nearest FTC (categories), whether household head received formal education, distance to nearest dry season road, distance to nearest all-weather road, distance to nearest marketplace, and distance to DA office/house. *** p<0.01, ** p<0.05, * p<0.1.

# Appendix H: Cost-effectiveness estimation procedures

To estimate the cost-effectiveness of the video-mediated approach, we measure the cost of an additional adoption that results from adding the video-mediated extension approach to the existing system. This is, in effect, a measure of marginal cost-effectiveness. We define the marginal cost-effectiveness ratio (MCER) as:

$${MCER}_{t}= \frac{c_{tp}}{n_{tp}}$$

where $c_{tp}$ measures the total costs of using video-mediated extension to promote the focal technologies in year *t* in the sample of *woredas* and *kebeles* assigned to the intervention *p*. The term $n_{tp}$ denotes the corresponding number of additional adopters, that is, those farmers who would not have adopted any of the focal technologies had there been no video-mediated extension approach. We detail below our estimation of these parameters.

Drawing on the cost-effectiveness analysis by Mogues et al. (2019), we focus on project-level costs associated with the video-mediated approach. Digital Green’s costs include personnel costs, training costs, operational costs, capital costs, and indirect costs incurred only in *kebeles* where video-mediated extension was conducted. We consider two cost scenarios: one which captures the marginal cost effectiveness of the intervention based on the coverage of the intervention associated with our RCT (the “Experimental Scenario”); and another which assumes that the intervention is targeted to all *kebeles* in the targeted *woredas*, and to all development groups in each kebele (a “Saturation Scenario”).

Table H1 summarizes all costs that we account for in this analysis. Several features of these costs are worth highlighting, as follows.

1. The costs analyzed here are the total costs incurred by Digital Green in promoting the video-mediated approach in this intervention over one *meher* season (2017/18), in 240 *kebeles* in 30 *woredas*.
2. Some costs are recurrent, such as personnel, operational, and indirect costs. For other costs such as equipment (computers, cameras, PICO projectors) and DA training costs, we assume a three-year lifespan, such that annualized costs represent one-third of the effective cost incurred during the one-year implementation of intervention.
3. Some costs are fixed at the *woreda*-level (see upper panel in Table H1). This implies that their contribution to the total cost is only affected by the number of *woredas*, not by the number of *kebeles* in each *woreda*. For example, extension staff need only one camera and one computer per *woreda* to produce and record videos, irrespective of whether one or all *kebeles* in a given *woreda* are targeted.
4. Some costs are fixed at the kebele-level (lower panel in Table H1). This means that their contribution to the total cost is affected by the number of *kebeles*, but not by the number of development groups targeted in each kebele. For example, extension staff need only one PICO projector per kebele to screen videos to farmers.

With regard to adoption, we take measure adoption of any of the three focus technologies promoted by the video-mediated extension approach.

With this in mind, we let $c_{tp}$ measure the yearly total costs of using video-mediated extension to promote the focal technologies in 240 *kebeles* in 30 *woredas*.

**Table H1. Annualized costs of the video-mediated intervention in 2017/18 meher**

| Costs | Experimental scenario | | Saturation scenario |
| --- | --- | --- | --- |
| Digital Green’s costs | Costs in RCT *kebeles* (USD) | Annualized costs (USD) | Annualized costs (USD) |
| *Woreda*-level costs |  |  |  |
| Personnel | 31,416 | 31,416 |  |
| Training | 137,645 | 45,882 |  |
| Operational | 32,933 | 32,933 |  |
| Laptop (30 - 1 per *woreda*) | 7,767 | 2,589 |  |
| Camera (30 - 1 per *woreda*) | 43,096 | 14,365 |  |
| Indirect costs | 76,782 | 76,782 |  |
| Total costs | 329,639 | 203,967 |  |
| *Kebele*-level costs |  |  |  |
| PICO (240 - 1 per kebele) | 52,235 | 17,412 |  |
| Total cost |  | 221,379 | 269,261 |

We measure the percent increase in the uptake rate of any of the focal technologies attributable to the video-mediated intervention using the coefficient $\beta$ on the treatment variable in the following specification:

$$y_{i}=\alpha+\beta T_{k}+X_{i}^{'}\delta+\pi_{w}+\varepsilon_{i}$$

where $y_{i}$ denotes the level of outcome$y$ measured at the household level $i$—whether or not the household has tried any of the focal technologies for any one of the three focus crops. The variable $T_{k}$ indicates the treatment status of *kebele* $k$ where the household lives (with a value of 1 if household was treated and 0 if control). $X$ is a vector of household- and development group-level characteristics that account for baseline imbalances between groups and augments the overall power of our estimates. We account for *woreda*-level stratification of our design through $\pi_{w}$, with a set of *woreda*-level fixed effects. We also account for treatment assignment at the *kebele* level by clustering our standard errors at that level.

Since we use intent-to-treat (ITT) impact estimates, the coverage area consists of the development groups and their members list of farmers targeted by Digital Green in the treatment group that were targeted by the intervention. If we let $N_{t}$ be the number of targeted households in the treatment *kebeles,* then the total number of additional adopters can be expressed as:

$$n_{tp}=\beta N_{t}$$

Corresponding estimates are provided in Table H2, under the column heading “Experimental scenario.”

**Table H2. Project coverage/reach and impact**

| Coverage | Experimental scenario | Saturation scenario |
| --- | --- | --- |
| Number of treated *kebeles* | 231 | 450 |
| Number of treated development groups per *kebele* | 10 | 30 |
| Number of treated farmers per development group | 25 | 25 |
| Number of farmers targeted | 57,750 | 337,500 |
| Impact estimates (ITT) on household head: | 0.062 | 0.062 |

The marginal cost-effectiveness ratio described above can now be calculated as:

$${MCER}_{t}= \frac{c_{tp}}{n_{tp}}=\frac{c_{tp}}{{\beta N}_{t}^{i}}$$

Table H3 report the marginal costs of adoption for each of the three technologies considered. Under the experimental scenario, the cost of each additional adoption of row planting was USD 30 (ETB 691). Similarly, for lower seeding rate and urea top/side dressing, the cost of each additional adoption was USD 16 (ETB 376) and USD 18 (USD 424), respectively.

In Table H3 we also report estimates of MCER under the saturation scenario. The corresponding increase in coverage is shown in column 3 of Table H2. On the cost side, as shown in column 4 of Table H1, those that are fixed at the woreda-level are not affected by the increase in per-woreda coverage. However, all kebele-level costs increase with the number of additional kebeles that are included. To account for the potential difficulties of reaching all development groups in a kebele with one PICO projector, we account for two PICO projectors per kebele in the saturation scenario.

Given the importance of fixed costs at the woreda level, the associated MCERs under full saturation are much lower than that of the experimental scenario. Securing one additional adoption of row planting costs just USD 6 (ETB 144); recommended seeding rates just USD 3 (ETB 78), and urea top/side dressing costs just USD 4 (ETB 88).

**Table H3. Marginal cost-effectiveness ratios in USD and ETB**

| **Technology** | **Experimental scenario** | | **Saturation scenario** | |
| --- | --- | --- | --- | --- |
|  | USD | ETB | USD | ETB |
| Row planting | 30 | 691 | 6 | 144 |
| Lower seeding rate | 16 | 376 | 3 | 78 |
| Urea top/side dressing | 18 | 424 | 4 | 88 |

***Note*: Calculated at the 2017 exchange rate of 23 ETB/USD.**
